# Hlf Expression Marks Early Emergence of Hematopoietic Stem Cell Precursors With Adult Repopulating Potential and Fate

**DOI:** 10.3389/fcell.2021.728057

**Published:** 2021-09-13

**Authors:** Wanbo Tang, Jian He, Tao Huang, Zhijie Bai, Chaojie Wang, Haizhen Wang, Ruichuang Yang, Yanli Ni, Jun Hou, Junliang Wang, Jie Zhou, Yingpeng Yao, Yandong Gong, Siyuan Hou, Bing Liu, Yu Lan

**Affiliations:** ^1^State Key Laboratory of Proteomics, Academy of Military Medical Sciences, Academy of Military Sciences, Beijing, China; ^2^Key Laboratory for Regenerative Medicine of Ministry of Education, School of Medicine, Institute of Hematology, Jinan University, Guangzhou, China; ^3^National Clinical Research Center for Infectious Diseases, Fifth Medical Center of Chinese PLA General Hospital, Beijing, China; ^4^State Key Laboratory of Experimental Hematology, Fifth Medical Center of Chinese PLA General Hospital, Beijing, China; ^5^Department of Radiotherapy, Fifth Medical Center of Chinese PLA General Hospital, Beijing, China

**Keywords:** pre-hematopoietic stem cells, hematopoietic stem cells, Hlf, aorta-gonad-mesonephros, genetic lineage tracing, hematopoietic development

## Abstract

In the aorta-gonad-mesonephros (AGM) region of mouse embryos, pre-hematopoietic stem cells (pre-HSCs) are generated from rare and specialized hemogenic endothelial cells (HECs) *via* endothelial-to-hematopoietic transition, followed by maturation into bona fide hematopoietic stem cells (HSCs). As HECs also generate a lot of hematopoietic progenitors not fated to HSCs, powerful tools that are pre-HSC/HSC-specific become urgently critical. Here, using the gene knockin strategy, we firstly developed an *Hlf-tdTomato* reporter mouse model and detected Hlf-tdTomato expression exclusively in the hematopoietic cells including part of the immunophenotypic CD45^–^ and CD45^+^ pre-HSCs in the embryonic day (E) 10.5 AGM region. By *in vitro* co-culture together with long-term transplantation assay stringent for HSC precursor identification, we further revealed that unlike the CD45^–^ counterpart in which both Hlf-tdTomato-positive and negative sub-populations harbored HSC competence, the CD45^+^ E10.5 pre-HSCs existed exclusively in Hlf-tdTomato-positive cells. The result indicates that the cells should gain the expression of Hlf prior to or together with CD45 to give rise to functional HSCs. Furthermore, we constructed a novel *Hlf-CreER* mouse model and performed time-restricted genetic lineage tracing by a single dose induction at E9.5. We observed the labeling in E11.5 AGM precursors and their contribution to the immunophenotypic HSCs in fetal liver (FL). Importantly, these Hlf-labeled early cells contributed to and retained the size of the HSC pool in the bone marrow (BM), which continuously differentiated to maintain a balanced and long-term multi-lineage hematopoiesis in the adult. Therefore, we provided another valuable mouse model to specifically trace the fate of emerging HSCs during development.

## Introduction

Hematopoietic stem cells (HSCs) are a unique cell population which can differentiate into nearly all kinds of blood lineage and maintain its quantity by self-renewal throughout the lifetime (Dzierzak and Bigas, 2018). At embryonic day (E) 10.5, the first functional HSCs are detected in the aorta-gonad-mesonephros (AGM) region (Muller et al., 1994; Medvinsky and Dzierzak, 1996; de Bruijn et al., 2000, 2002; North et al., 2002). At around E11.5, AGM HSCs start to migrate and colonize the fetal liver (FL) where they rapidly expand (Sánchez et al., 1996). During the perinatal period, HSCs begin to migrate to the bone marrow (BM) where they permanently reside throughout adult life (Morrison et al., 1995; Christensen et al., 2004).

It is generally accepted that HSCs are derived from specialized endothelial cells termed hemogenic endothelial cells (HECs) through endothelial-to-hematopoietic transition in mid-gestational embryos (Zovein et al., 2008; Chen et al., 2009; Dzierzak and Bigas, 2018; Neo et al., 2021). During this process, HECs bud from the dorsal aorta and then aggregate to form intra-aortic hematopoietic clusters (IAHCs) (Boisset et al., 2010). The earliest IAHCs appear at E9.5, and their number peaks to about 600 at E10.5 (Yokomizo and Dzierzak, 2010). IAHCs comprise progenitors that simultaneously express endothelial surface markers, such as CD31 and VE-Cadherin and hematopoietic marker Runx1 (Taoudi et al., 2008; Yamamoto et al., 2013; Solaimani Kartalaei et al., 2015). Pre-hematopoietic stem cells (pre-HSCs) are an intermediate population between HECs and HSCs (Hou et al., 2020), which do not have the capacity to repopulate irradiated recipients directly but can obtain this capacity after being co-cultured with stromal cells. Pre-HSCs are considered to be localized mainly within the IAHCs and reach approximately 50 at around E11 (Taoudi et al., 2008; Rybtsov et al., 2011, 2016; Boisset et al., 2015; Baron et al., 2018). These cells experience two stages including type I pre-HSCs (CD45^–^, known as T1 pre-HSCs) and type II pre-HSCs (CD45^+^, known as T2 pre-HSCs), which can both be detected in the E11.0 AGM region (Taoudi et al., 2008; Rybtsov et al., 2011; Zhou et al., 2016).

To isolate and decipher the embryonic hematopoietic populations, several researches have made great efforts in developing novel enrichment strategies or mouse models over the past few decades. With the help of the genetic lineage tracing mouse model *VE-Cadherin-Cre*, HSCs are proven to be derived from endothelial cells (Zovein et al., 2008; Chen et al., 2009). The progenies of endothelial cells contribute to a fraction of hematopoietic cells in adult BM, thymus, and spleen when labeled at E9.5 (Zovein et al., 2008; Chen et al., 2009). Additionally, time-lapse confocal imaging of *Ly6A-GFP* embryo slices confirms that aortic endothelial cells expressing GFP can generate Ly6A-GFP^+^Kit^+^CD41^+^ hematopoietic stem progenitor cells (HSPCs) at E10.5 (Boisset et al., 2010). The iterations of index-sorting analyses on *Gata2IRESVenus* reporter mice suggest that the functional HSCs emerge in the E11 IAHCs with a quantity of one to two cells (Vink et al., 2020). Recently, HECs are proven to be highly enriched by a cocktail of surface markers named PK44 (CD41^–^CD43^–^CD45^–^CD31^+^CD201^+^Kit^+^CD44^+^) or the newly established reporter *Neurl3-EGFP* (Hou et al., 2020). Previous studies verified T1 and T2 pre-HSCs exhibit phenotypes as VE-Cadherin^+^CD45^–^CD41^lo^ and VE-Cadherin^+^CD45^+^ in the E11.5 AGM region, respectively (Taoudi et al., 2008; Rybtsov et al., 2011). Notably, functional T1 and T2 pre-HSCs are identified to be highly enriched by CD201 and, thus, are further precisely characterized as CD31^+^CD45^–^CD41^lo^Kit^+^CD201^hi^ and CD31^+^CD45^+^Kit^+^CD201^hi^ at E11.0, respectively (Zhou et al., 2016).

Hlf is a transcription factor that belongs to the proline and acidic amino acid-rich (PAR) basic leucine zipper (bZip) family. Hlf is initially recognized in leukemia, and recent studies have identified its high expression in BM HSCs and essential role in maintaining hematopoiesis in a quiescent state (Gazit et al., 2013; Komorowska et al., 2017; Wahlestedt et al., 2017). In embryos, Hlf is specially expressed in IAHCs and FL HSCs without marking yolk sac erythro-myeloid progenitors, and its expression level is up-regulated along the maturation of HSCs during development (Yokomizo et al., 2019).

Recently, we identified *Hlf* as a signature gene for pre-HSCs in addition to HSCs (Zhou et al., 2016), suggesting that Hlf is a suitable molecule for studying the emergence of early pre-HSCs and their contribution to adult hematopoiesis. Therefore, we newly constructed *Hlf-tdTomato* and *Hlf-CreER* mouse models to investigate the dynamic marker changes of pre-HSCs and the adult fate of pre-HSCs labeled by *Hlf-CreER*.

## Materials and Methods

### Mice

Mice were fed at the Laboratory Animal Center of Academy of Military Medical Sciences in accordance with institutional guidelines. Mouse operations were approved by the Animal Care and Use Committee of the institute. The *Hlf^*tdTomato*/^*^+^ reporter mouse line and the *Hlf^*CreER*/^*^+^ lineage tracing mouse line were generated with the CRISPR/Cas9-mediated gene knockin technique by Beijing Biocytogen and Shanghai Model Organisms Center, respectively. The *ROSA^*ZsGreen*/ZsGreen^* reporter mice were described previously (Madisen et al., 2010). CD45.1/1 mice were purchased from Jackson Laboratory. CD45.2/2 mice were purchased from SPF (Beijing) Biotechnology Co., Ltd. All mice were maintained on C57BL/6 background. Embryos were staged by the number of somite pair (sp): E10.5, 36–40 sp; E11.0, 41–45 sp; and E11.5, 46–50 sp. The AGM region was dissected as previously described (Li et al., 2012).

### Flow Cytometry

Cells were analyzed and sorted by flow cytometers FACSymphony and FACS Aria 2 (BD Biosciences), respectively. Data were analyzed by FlowJo software (Tree Star). The antibodies with their supplier, clone number, dilution ratio, and catalog number are listed as follows: BD Biosciences: Rat anti-mouse Ly6A/E-BV605 (D7, 1:100, Cat#563288), Rat anti-mouse CD45-BV421 (30-F11, 1:40, Cat#563890), Rat monoclonal anti-mouse CD41-APC (MWReg30, 1:40, Cat#740903), Rat anti-mouse CD43-PE-Cy7 (S7, 1:50, Cat#562866), Rat anti-mouse CD31-BV786 (MEC13.3, 1:200, Cat#740870), and Rat anti-mouse TER-119-BV421 (TER-119, 1:40, Cat#566248); Biolegend: Rat monoclonal anti-mouse CD117 (c-kit)-BV650 (ACK2, 1:100, Cat#135125), Rat monoclonal anti-mouse CD150-BV785 (TC15-12F12.2, 1:100, Cat#115937), Rat monoclonal anti-mouse CD48-BV711 (HM48-1, 1:50, Cat#103439), Rat monoclonal anti-mouse CD16/32-BV510 (93, 1:50, Cat#101333), Rat monoclonal anti-mouse/human CD44-BV510 (IM7, 1:20, Cat#103044), Rat monoclonal anti-mouse CD144-BV421 (BV13, 1:50, Cat#138013), Rat monoclonal anti-mouse Ly6G-PE-Cy7 (1A8, 1:100, Cat#127618), and Mouse monoclonal anti-mouse CD45.2-PerCP5.5 (104, 1:100, Cat#109828); eBioscience: Rat monoclonal anti-mouse CD117 (c-kit)-APC-Cy7 (2B8, 1:200, Cat#47-1171-82), Rat monoclonal anti-mouse CD48-APC-Cy7 (HM48-1, 1:100, Cat#47-0481-82), Rat monoclonal anti-mouse CD34-eFluor 660 (RAM34, 1:20, Cat#50-0314-82), Rat monoclonal anti-mouse CD34-eFluor 450 (RAM34, 1:20, Cat#48-0341-82), Rat monoclonal anti-mouse CD11b-PE-Cy7 (M1/70, 1:200, Cat#25-0112-82), Rat monoclonal anti-mouse CD11b-FITC (M1/70, 1:400, Cat#11-0112-85), Rat monoclonal anti-mouse CD201-PE (eBio1560, 1:200, Cat#12-2012-80), Rat monoclonal anti-mouse CD201-APC (eBio1560, 1:100, Cat#17-2012-82), Rat monoclonal anti-mouse CD45-PE-Cy7 (30-F11, 1:200, Cat#25-0451-82), Rat monoclonal anti-mouse CD41-APC (eBioMWReg30, 1:100, Cat#17-0411-80), Rat monoclonal anti-mouse CD135-APC (A2F10, 1:20, Cat#17-1351-82), Rat monoclonal anti-mouse TER-119-PE-Cy7 (TER-119, 1:200, Cat#25-5921-82), Rat monoclonal anti-mouse Ly6G/Ly-6C-FITC (RB6-8C5, 1:200, Cat#11-5931-86), Rat monoclonal anti-mouse B220-BV421 (RA3-6B2, 1:40, Cat#48-0452-80), Rat monoclonal anti-mouse CD4-APC-Cy7 (GK1.5, 1:200, Cat#47-0041-82), Rat monoclonal anti-mouse CD8-PE-Cy7 (53-6.7, 1:40, Cat#25-0081-82), Rat monoclonal anti-mouse CD42d-APC (1C2, 1:40, Cat#17-0421-80), Rat monoclonal anti-mouse CD3e-APC (145-2C11, 1:200, Cat#17-0031-83), Mouse monoclonal anti-mouse CD45.1-APC (A20, 1:100, Cat#17-0453-82), Rat monoclonal anti-mouse Ly6G/Ly-6C-Biotin (RB6-8C5, 1:100, Cat#13-5931-82), Rat monoclonal anti-mouse TER-119-Biotin (TER-119, 1:100, Cat#13-5921-82), Rat monoclonal anti-mouse B220-Biotin (RA3-6B2, 1:400, Cat#13-0452-82), Rat monoclonal anti-mouse CD4-Biotin (RM4-5, 1:400, Cat#13-0042-82), Rat monoclonal anti-mouse CD8a-Biotin (53-6.7, 1:100, Cat#13-0081-81), Rat monoclonal anti-mouse CD11b-Biotin (M1/70, 1:400, Cat#13-0112-82), Rat monoclonal anti-mouse CD127-Biotin (A7R34, 1:100, Cat#13-1271-81), Streptavidin APC-eFluor 780 (1:50, Cat#47-4317-82), and Streptavidin eFluor 450 (1:50, Cat#48-4317-82).

### OP9-DL1 Co-culture and Transplantation Assay

The OP9-DL1 stromal cells were thawed 4 days before co-culture, and 3 × 10^4^ OP9-DL1 stromal cells (passage 3–7) were seeded into a 24-well plate the day before co-culture without irradiation. A total of 15 AGM regions were dissected and digested into single-cell suspension. The cells from AGM regions were stained with antibodies and sorted by flow cytometry with the gating strategies for indicated populations. Then, the sorted cells were seeded into a 24-well plate containing OP9-DL1 feeder cells with 3 ee (embryo equivalents) per well and incubated in α-MEM (Gibco) with 10% fetal bovine serum (Hyclone) and cytokines (100 ng/ml SCF, 100 ng/ml IL-3, and 100 ng/ml Flt3 ligand, PeproTech) for 6 days. On the fourth day, a half amount of cell culture medium mentioned above was added to the wells. Six days later, the co-cultured cells in each well were detached by 0.25% trypsin (Beyotime Biotechnology, C0203) and harvested independently for flow cytometry analysis or transplantation assay. For transplantation, male CD45.1/1 were mated with female *Hlf^*tdTomato*/^*^+^ (CD45.2/2) to obtain CD45.1/2 embryos. Cell suspension harvested after 6 days of co-culture were mixed with 2 × 10^4^ nucleated BM helper cells (CD45.2/2). The mixture was injected *via* the tail vein into 8–12-week-old female recipients (CD45.2/2) which had been exposed to a split dose of 9 Gy γ-irradiation (^60^Co). The recipients demonstrating ≥5% donor-derived chimerism in peripheral blood were considered as successfully reconstituted. Donor contribution to each blood lineage is calculated as: (percentage of donor cells of a given lineage in CD45^+^ cells/total percentage of a given lineage in CD45^+^ cells) × 100%; Donor contribution to LSK (Lin^–^Sca1^+^Kit^+^) cells is calculated as: percentage of donor cells in LSK cells × 100% (Benz et al., 2012; Li et al., 2012; Ye et al., 2017).

### Immunofluorescence

Embryos were isolated and fixed with 4% paraformaldehyde for 2–4 h at 4°C, embedded in paraffin and sectioned into 5-μm slices using Leica RM2235. Sections were placed in an oven at 60°C for 15 min, deparaffinized with ethanol of gradient concentration, and subsequently placed in a microwave for 20 min at 95°C within citrate buffer (pH 6.0). The sections were cooled to room temperature and then placed in 3% H_2_O_2_ for 20 min to remove endogenous peroxidase. Sections were blocked in blocking solution (1:1, Cat#ZLI-9056, Zhongshan golden bridge) for 30 min at room temperature, and then incubated with primary antibodies overnight at 4°C. After being washed with PBS three times, sections were incubated with corresponding secondary antibodies (Zhongshan golden bridge) for 30 min at room temperature. After being washed with PBS, sections were stained with DendronFluor TSA (Histova, NEON 4-color IHC Kit for FFPE, NEFP450, 1:100, 20–60 s). After the staining of the first antigen was completed, we thoroughly eluted the primary and secondary antibodies by re-heating the slides in a microwave with citrate buffer (pH 6.0) for 20 min at 95°C, and repeated the above steps from the microwave repair step to the DendronFluor TSA staining step, so that each antigen was labeled by distinct fluorophores finally. After all the antibodies were stained, the slices were stained with DAPI (4’,6-diamidino-2-phenylindole). Images were collected by confocal microscope (Nikon Ti-E A1/ZEISS LSM 880). The primary antibodies and their clone number, dilution ratio, catalog number, and company are listed as follows: Rabbit polyclonal anti-RFP (1:1,500, Cat#600-401-379, Rockland); Rabbit monoclonal anti-Estrogen Receptor alpha (SP1, 1:200, Cat#ab16660, Abcam), Rabbit monoclonal anti-RUNX1/AML1 + RUNX3 + RUNX2 (EPR3099, 1:150, Cat#ab92336, Abcam), and Rabbit monoclonal anti-CD31 (EPR17259, 1:1,000, Cat#ab182981, Abcam). The second antibody was HRP-labeled Goat anti-Rabbit IgG polymer (1:1, Cat#PV6001, Zhongshan golden bridge).

### Inducible Genetic Lineage Tracing

Tamoxifen free base (T5648-5G, Sigma) 200 mg was dissolved in 10 ml sunflower-seed oil (S5007; Sigma) and shaken overnight at the room temperature. For lineage tracing, *Hlf*^*CreER/*+^ mice were firstly bred with *ROSA*^*ZsGreen/ZsGreen*^ mice to generate *Hlf*^*CreER/*+^*; ROSA^*ZsGreen/*^*^+^ (referred as *Hlf-CreER;ZsGreen*), then male *Hlf-CreER;ZsGreen* mice were crossed with female C57BL/6 mice. The pregnant C57BL/6 mice were administered with a single dose of tamoxifen by gavage (3 mg per 30 g body weight) at E9.5. At indicated embryonic stages, the pregnant C57BL/6 mice were sacrificed, the embryos were isolated with subsequent genotyping, and the *Hlf-CreER;ZsGreen* embryos were used for further flow cytometry analysis, with the *ROSA*^*ZsGreen/*+^ embryos from the same litter serving as controls. For adult mice analysis, *Hlf-CreER;ZsGreen* offspring with a single dose of tamoxifen induction at E9.5 as described above were used for flow cytometry analysis.

### Single Cell RNA-Seq Dataset Source

The scRNA-seq data are from our previous study (Zhou et al., 2016) and downloaded from the GEO database (GSE67120).

## Results

### Establishment of an *Hlf-tdTomato* Reporter Mouse Model

**FIGURE 1:**
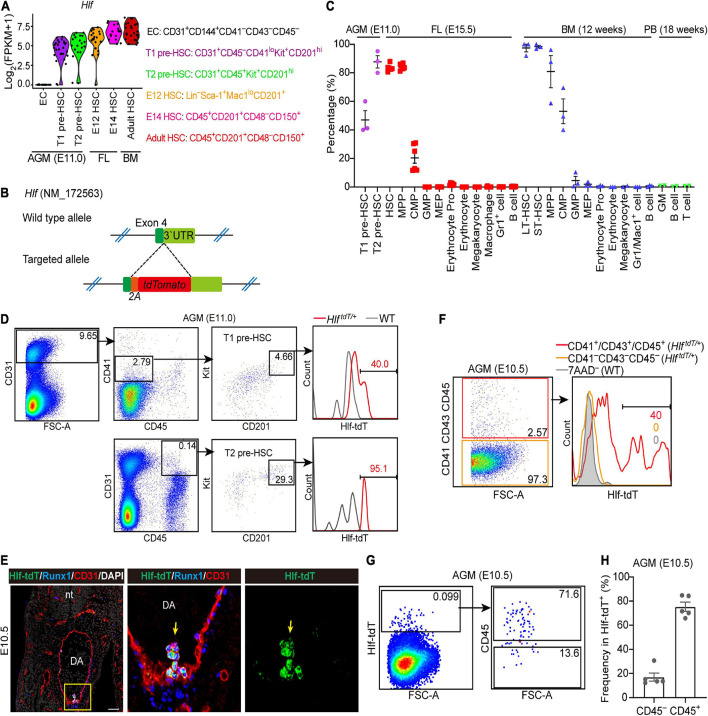
Establishment of an *Hlf-tdTomato* reporter mouse model. **(A)** Violin plots showing the transcription level of *Hlf* in six populations as indicated. Data are from GSE67120. **(B)** Schematic model of the gene-targeting strategy for constructing *Hlf-tdTomato* reporter mouse line *via* CRISPR/Cas9 system. **(C)** Graph showing the percentage of Hlf-tdTomato expression in each hematopoietic population of the E11.0 AGM region, E15.5 fetal liver (FL), adult bone marrow (BM), and peripheral blood (PB). Data are shown as means ± SEM from at least two independent experiments. MPP, multipotent progenitor; CMP, common myeloid progenitor; GMP, granulocyte-monocyte progenitor; MEP, megakaryocyte-erythroid progenitor; erythrocyte pro, erythrocyte progenitor; LT-HSC, long-term hematopoietic stem cell; ST-HSC, short-term hematopoietic stem cell. **(D)** Representative FACS plots showing the Hlf-tdTomato expression in T1 and T2 pre-HSCs in the AGM region of E11.0 *Hlf-tdTomato* embryos. Data are representative of three independent experiments. *Hlf*^*tdT/*+^, *Hlf^*tdTomato*/^*^+^; WT, wild type. **(E)** Representative immunostaining on sections from the AGM region of E10.5 *Hlf-tdTomato* embryos. Arrows indicate Hlf-tdTomato^+^ IAHCs. The two images to the right present the high magnification view of yellow box. nt, neural tube; DA, dorsal aorta. Scale bar, 50 μm. **(F)** Representative FACS plots showing the proportion of Hlf-tdTomato positive cells in CD41^–^CD43^–^CD45^–^ and CD41^+^/CD43^+^/CD45^+^ populations in the AGM region of E10.5 *Hlf-tdTomato* embryos. Data are representative of two independent experiments. Genotypes are indicated in the brackets. *Hlf*^*tdT/*+^, *Hlf^*tdTomato*/^*^+^; WT, wild type. **(G)** Representative FACS plots showing the CD45 expression in Hlf-tdTomato^+^ cells in the AGM region of E10.5 *Hlf-tdTomato* embryos. **(H)** Graph showing the frequencies of CD45^–^ or CD45^+^ cells in Hlf-tdTomato^+^ cells of the E10.5 AGM region. Data are shown as means ± SEM from five independent experiments.

By analyzing the transcriptomic dataset we previously constructed (Zhou et al., 2016), we found that *Hlf* was expressed in almost all of the HSC-competent cells from embryo to adult but not in endothelial cells of the mid-gestational AGM region, with a gradual up-regulation from pre-HSCs to adult HSCs (Figure 1A). Therefore, for a better understanding about the stepwise specification of nascent HSCs, we generated an *Hlf-tdTomato* reporter mouse model using the gene knockin strategy to insert a P2A-tdTomato cassette between exon 4 and stop codon of *Hlf* gene locus without disrupting its expression (Figure 1B).

We firstly evaluated the expression pattern of Hlf-tdTomato in hematopoietic populations of the E11.0 AGM region, E15.5 FL, and adult BM and peripheral blood by fluorescence activated cell sorting (FACS) analyses. With the use of the highly functionally enriched markers of pre-HSCs (Zhou et al., 2016), we showed that about half of the immunophenotypic T1 pre-HSCs and most if not all of T2 pre-HSCs at E11.0 were positive for Hlf-tdTomato (Figures 1C,D). Furthermore, Hlf-tdTomato was expressed in more than 80% of immunophenotypic HSCs and multi-potent progenitors, much higher than in common myeloid progenitors, but was seldom expressed in other committed progenitors and mature blood cells in E15.5 FL (Figure 1C and Supplementary Figure 1A). In adult BM, Hlf-tdTomato was expressed in almost all the immunophenotypic long-term HSCs and short-term HSCs, irrespective of the marker combinations used, and also in a predominant portion of multi-potent progenitors and in about half of common myeloid progenitors, but hardly in other progenitors and mature blood cells (Figure 1C and Supplementary Figures 1B,C). Analysis of peripheral blood showed that mature blood lineages, including granulocytes/monocytes, B cells, and T cells, lacked Hlf-tdTomato expression (Figure 1C and Supplementary Figure 1D). The expression pattern of Hlf-tdTomato in various hematopoietic populations at different developmental stages was not only basically in accordance with our transcriptomic data (Figure 1A) but also principally in line with a previous report (Yokomizo et al., 2019) validating the successful construction of the *Hlf-tdTomato* reporter mouse model.

We next investigated the expression of Hlf-tdTomato at E10.5 by immunostaining and flow cytometry. Immunostaining on sections of the E10.5 AGM region showed that Hlf-tdTomato was expressed in the IAHC cells, co-expressing hematopoietic transcription factor Runx1 and endothelial marker CD31, in line with a previous report (Yokomizo et al., 2019). On the other hand, Hlf-tdTomato expression was not detected in the endothelial layer of the dorsal aorta (Figure 1E). Consistently, FACS analyses indicated that Hlf-tdTomato expression was not detected in the immunophenotypic non-hematopoietic cells (CD41^–^CD43^–^CD45^–^) from multiple intra-embryonic sites, including the AGM region, head, trunk, and limb. In contrast, its expression was confined to the immunophenotypic hematopoietic cells (CD41^+^/CD43^+^/CD45^+^) (Figure 1F and Supplementary Figures 2A,B). We also noticed that only 0.069 ± 0.013% cells in the E10.5 AGM region expressed Hlf-tdTomato, in which 17.0 ± 3.4% cells were CD45-negative (Figures 1G,H). This expression pattern suggested that Hlf-tdTomato expression may initiate from CD45^–^ pre-HSCs along HSC specification.

### Hlf-tdTomato Expression in Functional CD45^–^ Pre-HSCs in the E10.5 AGM Region

**FIGURE 2:**
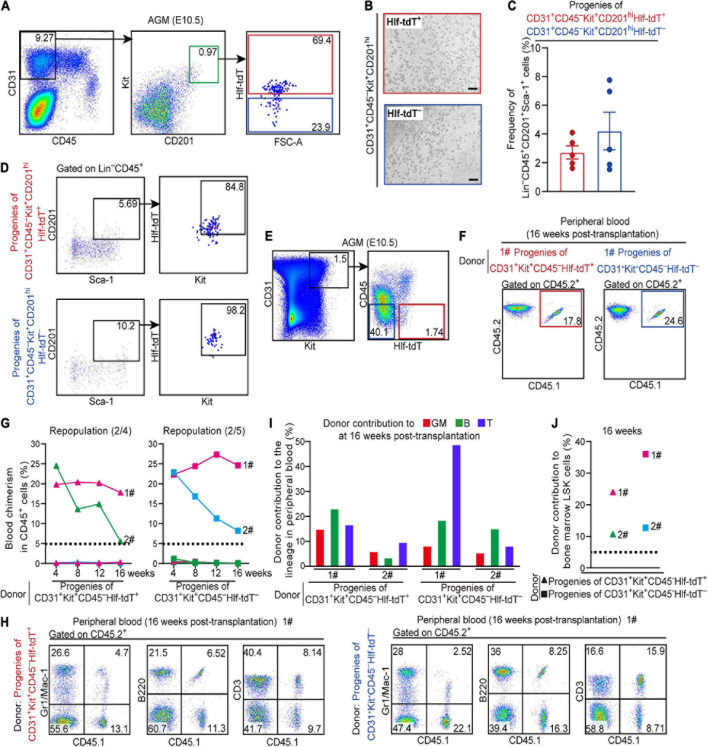
Hlf-tdTomato expression in functional CD45^–^ pre-HSCs in the E10.5 AGM region. **(A)** Representative FACS plots showing the proportions of Hlf-tdTomato^+^ and Hlf-tdTomato^–^ cells in CD31^+^CD45^–^Kit^+^CD201^hi^ population in the AGM region of E10.5 *Hlf-tdTomato* embryos. Data are representative of five independent experiments. **(B)** Representative morphology of hematopoietic cells generated from Hlf-tdTomato^+^ cells (red) and Hlf-tdTomato^–^ cells (blue) within CD31^+^CD45^–^Kit^+^CD201^hi^ population. Scale bar, 100 μm. **(C)** Graph showing the frequency of Lin^–^CD45^+^CD201^+^Sca-1^+^ cells generated from the indicated populations from the E10.5 AGM region after co-culture analyzed by FACS. Data are shown as means ± SEM from five independent experiments. **(D)** Representative FACS plots showing the expression of Hlf-tdTomato and Kit in Lin^–^CD45^+^CD201^+^Sca-1^+^ cells that are generated from the indicated populations of the E10.5 AGM region after co-culture. Data are representative of five independent experiments. **(E)** Sorting strategy for the populations from the AGM region of E10.5 *Hlf-tdTomato* embryos for co-culture/transplantation assay. The cell populations sorted for functional assay are denoted as colored boxes. **(F)** Representative FACS plots of blood chimerism of donor-derived (CD45.1^+^CD45.2^+^) cells in CD45^+^ cells at 16 weeks post-transplantation. **(G)** Graphs showing the blood chimerism in CD45^+^ cells of each of all the recipients from 4 to 16 weeks post-transplantation. The recipients were transplanted with the culture products of CD31^+^Kit^+^CD45^–^Hlf-tdTomato^+^ (triangle, left) or CD31^+^Kit^+^CD45^–^Hlf-tdTomato^–^ (square, right) cells from the AGM region of E10.5 *Hlf-tdTomato* embryos. Numbers of repopulated/total recipients are shown in the brackets. For each transplantation assay, cells from a total of 15 Hlf-tdTomato positive embryos belonging to three independent litters (biological repeats) were sampled and cultured as donor cells, and five recipients were used. Each line represents a recipient and each color represents an independent litter (biological repeat). **(H)** Representative FACS plots showing proportions of donor cells and each hematopoietic lineage at 16 weeks post-transplantation. Myeloid cells (Gr1/Mac-1^+^), B cells (B220^+^), and T cells (CD3^+^) in peripheral blood are shown. **(I)** Bar plot representing the percentages of donor contribution to granulocytes/monocytes (GM, red), B cells (green), and T cells (blue) in the peripheral blood at 16 weeks post-transplantation. **(J)** Graph showing donor contribution to the bone marrow hematopoietic stem progenitor cells (Lin^–^Sca-1^+^Kit^+^, LSK) of the successfully repopulated recipients at 16 weeks post-transplantation.

Previous studies showed that precursors of HSCs in E10.5 AGM are enriched in VE-cad^+^CD45^–^ population and CD201^hi^ population (Rybtsov et al., 2011; Hadland et al., 2017). We examined the E10.5 AGM region by FACS and found that Hlf-tdTomato expression was observed in 68.9 ± 3.6% of the CD31^+^CD45^–^Kit^+^CD201^hi^ population that has been proven to enrich functional T1 pre-HSCs (Figure 2A). Subsequently, we sorted Hlf-tdTomato^+^ and Hlf-tdTomato^–^ cells within the CD31^+^CD45^–^Kit^+^CD201^hi^ population from the E10.5 AGM region, respectively, and co-cultured them with OP9-DL1 *in vitro*. After 6 days of co-culture, both Hlf-tdTomato^+^ and Hlf-tdTomato^–^ groups generated typical hematopoietic clusters (Figure 2B). Progenies of both groups contained HSC-like cells with a Lin^–^CD45^+^Sca-1^+^CD201^+^ immunophenotype by FACS analysis (Hadland et al., 2017; Figure 2C). Moreover, most of these HSC-like cells expressed Hlf-tdTomato as well as Kit, further suggesting their HSC-like identity (Figure 2D).

For *in vivo* functional validation, Hlf-tdTomato^+^ and Hlf-tdTomato^–^ cells within CD31^+^Kit^+^CD45^–^ population from the E10.5 AGM region were, respectively, co-cultured with OP9-DL1 for 6 days, then the culture products were transplanted into lethally irradiated adult mice. At 16 weeks post-transplantation, multi-lineage repopulation was observed in peripheral blood and multiple organs (BM, spleen, and thymus) of the recipients of both Hlf-tdTomato^+^ (2/4) and Hlf-tdTomato^–^ (2/5) groups. Moreover, the contribution of the donor cells to the immunophenotypic HSCs and hematopoietic progenitors in BM was also observed, validating the bona fide HSC competence (Figures 2E–J and Supplementary Figures 3A,B). These data indicated that precursors of HSCs were distributed in both Hlf-tdTomato^+^ and Hlf-tdTomato^–^ subpopulations of CD45^–^ cells in the E10.5 AGM region. Considering the restricted expression of Hlf-tdTomato in the hematopoietic cells (Figure 1F), the CD45^–^Hlf-tdTomato^+^ population with the HSC-competence should belong to T1 pre-HSCs. On the other hand, as HSC-competent HECs in the AGM region represented by PK44 did not express Hlf-tdTomato (Supplementary Figure 2B), the repopulating capacity of the progenies of CD31^+^Kit^+^CD45^–^Hlf-tdTomato^–^ population should be partially due to the existence of HSC-primed HECs therein, although the existence of Hlf-tdTomato^–^ pre-HSCs could not be completely excluded.

### Hlf-tdTomato Enriches Functional CD45^+^ Pre-HSCs in the E10.5 AGM Region

**FIGURE 3:**
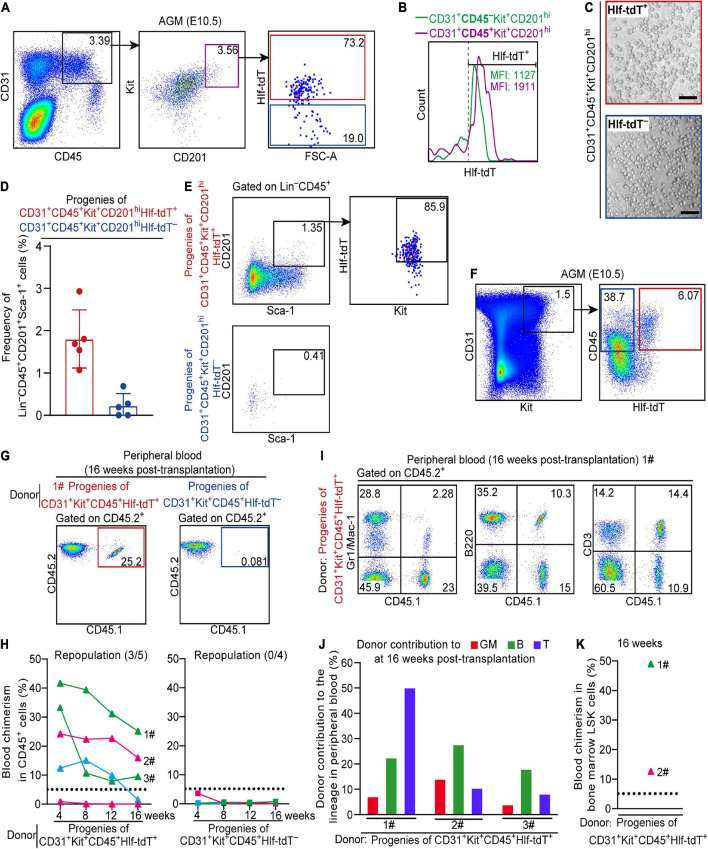
Hlf-tdTomato enriches functional CD45^+^ pre-HSCs in the E10.5 AGM region. **(A)** Representative FACS plots showing the proportion of Hlf-tdTomato^+^ and Hlf-tdTomato^–^ cells in the CD31^+^CD45^+^Kit^+^CD201^hi^ population in the AGM region of E10.5 *Hlf-tdTomato* embryos. Data are representative of five independent experiments. **(B)** Representative FACS histogram showing the fluorescence intensity of tdTomato in the CD31^+^CD45^–^Kit^+^CD201^hi^ and CD31^+^CD45^+^Kit^+^CD201^hi^ populations in the AGM region of E10.5 *Hlf-tdTomato* embryos. Values of mean fluorescence intensity of tdTomato in the Hlf-tdTomato^+^ cells are indicated. Data are representative of five independent experiments. **(C)** Representative morphology of hematopoietic cells generated from Hlf-tdTomato^+^ cells and Hlf-tdTomato^–^ cells within the CD31^+^CD45^+^Kit^+^CD201^hi^ population. Scale bar, 100 μm. **(D)** Graph showing the frequency of Lin^–^CD45^+^CD201^+^Sca-1^+^ cells generated from the indicated populations from the E10.5 AGM region after co-culture measured by FACS analysis. Data are shown as means ± SEM from five independent experiments. **(E)** Representative FACS plots showing the expression of Hlf-tdTomato and Kit in Lin^–^CD45^+^CD201^+^Sca-1^+^ cells that are generated from the indicated populations of the E10.5 AGM region after co-culture. Data are representative of five independent experiments. **(F)** Cell sorting strategy for the populations from the AGM region of E10.5 *Hlf-tdTomato* embryos for co-culture/transplantation assay. The cell populations sorted for functional assay are denoted by colored boxes. **(G)** Representative FACS plots of blood chimerism of donor-derived (CD45.1^+^CD45.2^+^) cells in CD45^+^ cells at 16 weeks post-transplantation. **(H)** Graphs showing the blood chimerism in CD45^+^ cells of each of all the recipients from 4 to 16 weeks post-transplantation. The recipients were transplanted with the culture products of CD31^+^Kit^+^CD45^+^Hlf-tdTomato^+^ (triangle, left) or CD31^+^Kit^+^CD45^+^Hlf-tdTomato^–^ (square, right) cells from the AGM region of E10.5 *Hlf-tdTomato* embryos. Numbers of repopulated/total recipients are shown in the brackets. For each transplantation assay, cells from a total of 15 Hlf-tdTomato positive embryos belonging to three independent litters (biological repeats) were sampled and cultured as donor cells, and five recipients were used. Each line represents a recipient and each color represents an independent litter (biological repeat). **(I)** Representative FACS plots showing proportions of donor cells and each hematopoietic lineage at 16 weeks post-transplantation. Myeloid cells (Gr1/Mac-1^+^), B cells (B220^+^), and T cells (CD3^+^) in peripheral blood are shown. **(J)** Bar plot showing the percentages of donor contribution to granulocytes/monocytes (GM, red), B cells (green), and T cells (blue) in the peripheral blood at 16 weeks post-transplantation. **(K)** Graph showing donor contribution to the bone marrow hematopoietic stem progenitor cells (Lin^–^Sca-1^+^Kit^+^, LSK) of two successfully repopulated recipients at 16 weeks post-transplantation. Next, Hlf-tdTomato^+^ and Hlf-tdTomato^–^ cells within the CD31^+^Kit^+^CD45^+^ population from the E10.5 AGM region were, respectively, isolated and co-cultured with OP9-DL1 for 6 days, then transplantation assay was performed with the culture products. Of note, multi-lineage repopulation in peripheral blood and multiple organs (BM, spleen, and thymus) at 16 weeks post-transplantation was only detected in the recipients of Hlf-tdTomato^+^ group, consistent with the detection of the chimerism in the immunophenotypic HSCs and hematopoietic progenitors in BM (Figures 3F–K and Supplementary Figures 3C,D). Therefore, Hlf-tdTomato expression further enriched the HSC-competence in E10.5 CD45^+^ population. The finding also indicated that if a cell expresses CD45 prior to Hlf at this early stage, it has lost the possibility of specification toward HSCs.

We noticed that the proportion of Hlf-tdTomato-expressing cells in the immunophenotypic T2 pre-HSCs (CD31^+^CD45^+^Kit^+^CD201^hi^) in the E10.5 AGM region was lower than that in the E11.0 AGM region (Figures 1D, 3A). Furthermore, the mean fluorescence intensity (MFI) of tdTomato in Hlf-tdTomato^+^ cells was higher in the CD45^+^ sub-population than in the CD45^–^ counterpart within CD31^+^Kit^+^CD201^hi^ cells (Figure 3B). As a previous report revealed that the expression intensity of Hlf gradually increases along with the maturation of embryonic HSCs (Yokomizo et al., 2019), we wondered whether *Hlf* expression marks the HSC-competence in the CD45^+^ cells as early as E10.5. Therefore, we sorted Hlf-tdTomato^+^ and Hlf-tdTomato^–^ within the CD31^+^CD45^+^Kit^+^CD201^hi^ population from the E10.5 AGM region (Figure 3A), and co-cultured them with OP9-DL1 *in vitro*, respectively. Six days later, both Hlf-tdTomato^+^ and Hlf-tdTomato^–^ groups generated hematopoietic clusters (Figure 3C), but the former yielded much more HSC-like cells (Lin^–^CD45^+^Sca-1^+^CD201^+^) than the latter by FACS analyses (Figures 3D,E).

### Generation of an Inducible *Hlf-CreER* Mouse Model

**FIGURE 4:**
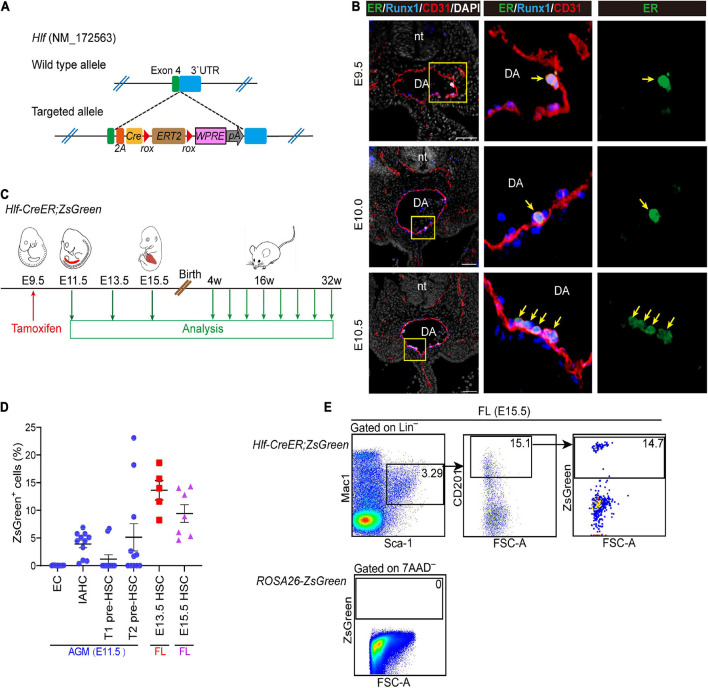
Generation of an inducible *Hlf-CreER* mouse model. **(A)** Schematic model of the gene-targeting strategy for constructing *Hlf-CreER* lineage tracing mouse line *via* CRISPR/Cas9 system. **(B)** Representative immunostaining on sections at the E9.5, E10.0, and E10.5 AGM region of *Hlf-CreER* embryos. Arrows indicate ER^+^ IAHCs. The images to the right present the magnification view of yellow boxes. Scale bar, 50 μm. **(C)** Schematic of the experimental design for the genetic lineage tracing with *Hlf-CreER;ZsGreen* mice. Single dose of induction by tamoxifen injection was performed at E9.5 (red arrow). Green arrows indicate the time points for analyses. The contribution of labeled cells in peripheral blood was chased every 4 weeks for up to 32 weeks after birth. **(D)** Graph showing the labeled proportions in the indicated populations. EC, endothelial cell. Data are shown as means ± SEM from three independent experiments. **(E)** Representative FACS plots showing the proportion of labeled cells in the immunophenotypic HSCs (Lin^–^Sca-1^+^Mac-1^lo^CD201^+^) in E15.5 fetal liver (FL). Data are representative of four independent experiments.

To determine the physiological contribution of the Hlf-expressing early cells, including pre-HSCs and nascent HSCs, to FL and adult hematopoiesis, we newly generated an *Hlf-CreER* mouse model by CRISPR/Cas9-mediated gene knockin strategy, with an inducible *CreERT2* cassette inserted between exon 4 and 3′UTR of the *Hlf* gene locus (Figure 4A). Immunostaining of ER in the AGM region showed the expression of the inducible Cre recombinase specifically in the IAHC cells from E9.5 to E10.5 (Figure 4B), consistent with the expression pattern of Hlf-tdTomato (Figure 1E).

Next, *Hlf-CreER;ROSA-LSL-ZsGreen* (referred to as *Hlf-CreER;ZsGreen*) mice were generated for lineage tracing. We performed the single-dose tamoxifen induction at E9.5, 24 h before the first HSCs appear at E10.5 (Figure 4C), and the labeling would involve the pre-HSCs and emerging HSCs marked by Hlf expression mainly within the next 48 h from injection (Zovein et al., 2008). We first assessed the labeling proportions of several populations related to HSC ontogeny by FACS analyses, including endothelial cells, IAHC cells, as well as T1 and T2 pre-HSCs in the E11.5 AGM region. Endothelial cells were not labeled as expected (Figure 4D and Supplementary Figure 4A). In contrast, the labeling was found in 3.9 ± 0.7% of the immunophenotypic IAHC cells (CD31^+^Kit^hi^) (Figure 4D and Supplementary Figure 4A). Compared with that in T1 pre-HSCs (CD31^+^CD45^–^CD41^lo^Kit^+^CD201^hi^, 1.2 ± 0.8%), the average constitution of labeled cells in T2 pre-HSCs (CD31^+^CD45^+^Kit^+^CD201^hi^) was higher (5.1 ± 2.5%) (Figure 4D and Supplementary Figure 4A), in line with the higher expression of Hlf-tdTomato in T2 pre-HSCs than in T1 pre-HSCs (Figure 1D). Interestingly, the labeling of pre-HSCs in individual AGM regions was concentrated in either T1 or T2 pre-HSCs (Supplementary Figure 4B), suggesting the transient dynamics of pre-HSC maturation.

We then evaluated the contribution of these Hlf-labeled early cells to the immunophenotypic HSCs in FL. Of note, the average constitution of lineage-labeled cells in FL HSCs (Lin^–^Sca-1^+^Mac-1^lo^CD201^+^) (Zhou et al., 2016) was 13.6 ± 1.8% at E13.5 and 9.4 ± 1.6% at E15.5, higher than that in pre-HSCs in the E11.5 AGM region (Figures 4D,E). When using another surface marker combination ESLAM (CD45^+^CD201^+^CD150^+^CD48^–^) (Benz et al., 2012) to check E15.5 FL HSCs, we came to a similar conclusion (Supplementary Figure 4C). These results indicated that *Hlf-CreER;ZsGreen* was able to trace part of AGM pre-HSCs and/or nascent HSCs without labeling endothelial cells when induced at E9.5, and the progenies of the labeled cells could migrate and colonize FL, possibly with an expansion to some extent.

### Hlf-Labeled Early Cells Contribute to Long-Term and Multi-Lineage Adult Hematopoiesis

**FIGURE 5:**
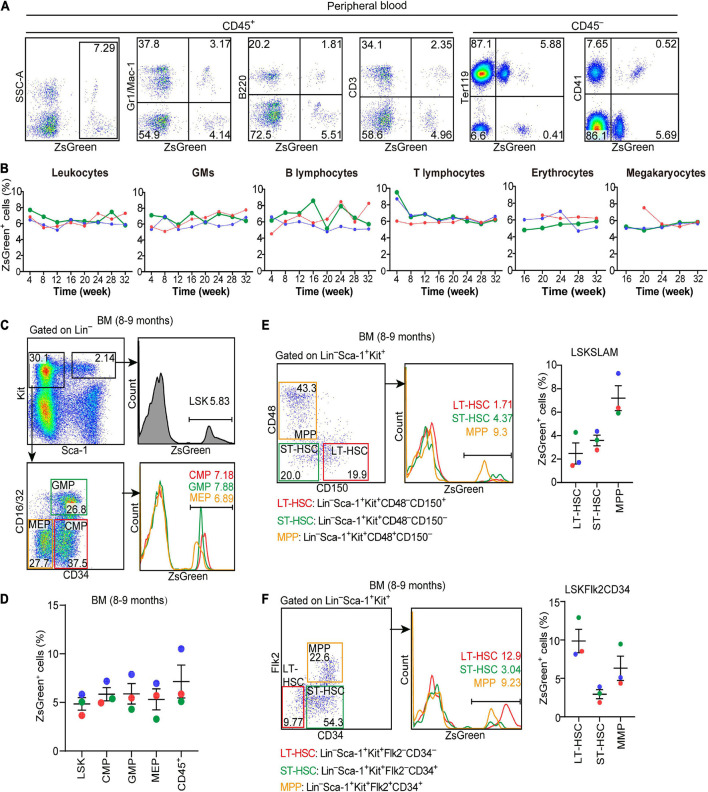
Hlf-labeled early cells contribute to long-term and multi-lineage adult hematopoiesis. **(A)** Representative FACS plots showing the proportions of Hlf-labeled cells in CD45^+^ cells and in each hematopoietic lineage in the peripheral blood from a 32-week old *Hlf-CreER;ZsGreen* mouse with tamoxifen administration at E9.5. **(B)** Graphs showing the dynamics of the labeling proportions in CD45^+^ leukocytes, granulocytes/monocytes (GMs), B lymphocytes, T lymphocytes, erythrocytes, and megakaryocytes, respectively, in the peripheral blood of *Hlf-CreER;ZsGreen* mice at 4–32 weeks old with tamoxifen administration at E9.5. Data are from three individuals represented by different colors. **(C)** Representative FACS plots showing the proportions of Hlf-labeled cells in LSK (Lin^–^Sca-1^+^Kit^+^) cells, CMPs, GMPs, and MEPs in the bone marrow (BM) of 8–9 month-old *Hlf-CreER;ZsGreen* mice with tamoxifen administration at E9.5. **(D)** Graph showing the proportions of Hlf-labeled cells in LSK cells, CMPs, GMPs, MEPs, and CD45^+^ cells in the bone marrow (BM) of 8–9 month-old *Hlf-CreER;ZsGreen* mice with tamoxifen administration at E9.5. Data are shown as means ± SEM from three independent experiments represented by different colors. CMP, common myeloid progenitor; GMP, granulocyte-monocyte progenitor; MEP, megakaryocyte-erythroid progenitor. **(E)** Representative FACS plots (left) and graph (right) showing the proportions of Hlf-labeled cells in LT-HSCs, ST-HSCs, and MPPs in the bone marrow (BM) of adult *Hlf-CreER;ZsGreen* mice with tamoxifen administration at E9.5. LSKSLAM marker combination was used. Data are from three independent experiments represented by different colors. **(F)** Representative FACS plots (left) and graph (right) showing the proportions of Hlf-labeled cells in LT-HSCs, ST-HSCs, and MPPs in the bone marrow (BM) of adult *Hlf-CreER;ZsGreen* mice with tamoxifen administration at E9.5. LSKFlk2CD34 marker combination was used. Data are from three independent experiments represented by different colors. LT-HSC, long-term hematopoietic stem cell; ST-HSC, short-term hematopoietic stem cell; MPP, multipotent progenitor.

To evaluate the contribution of Hlf-labeled early cells to adult hematopoiesis, we administrated a single dose of tamoxifen at E9.5, and the contribution to multiple hematopoietic lineages in peripheral blood was chased up to 32 weeks after birth (Figure 4C). The labeling in total CD45^+^ leukocytes was ranged from 5.2 to 7.7%, with little fluctuation from 4 to 32 weeks (Figures 5A,B). The constitutions of traced cells in different lineages were similar, including granulocytes/monocytes (CD45^+^Gr1/Mac-1^+^), B lymphocytes (CD45^+^B220^+^), T lymphocytes (CD45^+^CD3^+^), erythrocytes (CD45^–^Ter119^+^), and megakaryocytes (CD45^–^CD41^+^), with on average of 5.2–8.1% for all the lineages and all the time points detected (Figures 5A,B).

To further determine whether and to what extent the Hlf-labeled early cells contributed to BM hematopoiesis in adult, we analyzed BM samples from three 8–9-month-old mice by flow cytometry. The constitutions of lineage-labeled cells in the immunophenotypically defined committed hematopoietic progenitors, including common myeloid progenitors, granulocyte-monocyte progenitors, and megakaryocyte-erythroid progenitors, were similar to each other, and also to that in the upstream HSC-enriched LSK (Lin^–^Sca1^+^Kit^+^) cells as well as to those in the mature blood lineages in the peripheral blood (Figures 5C,D). Interestingly, when using different marker combinations to define the enriched long-term HSCs, short-term HSCs, and multi-potent progenitors, the results varied regarding the contribution of lineage-traced cells to each of these HSPC populations (Figures 5E,F), suggestive of the heterogeneity within these immunophenotypically defined HSPC populations.

Taken together, these data unambiguously indicated that the early Hlf-expressing cells in the embryo contribute to and retain the size of the HSC pool in the BM, which continuously differentiate to maintain a balanced and long-term multi-lineage hematopoiesis in the adult. Therefore, here we provided a valuable mouse model to specifically trace the fate of emerging HSCs in the embryo.

## Discussion

Considering that *Hlf* is an HSC-related gene that is specifically expressed in both embryonic and adult HSCs but neither in primitive blood cells nor erythro-myeloid progenitors, which is conserved in both mouse and human (Shojaei et al., 2005; Magnusson et al., 2007; Yokomizo et al., 2019; Zeng et al., 2019), here we specifically chose it and successfully constructed two mouse models, *Hlf-tdTomato* reporter mice and *Hlf-CreER* lineage tracing mice, which would without doubt serve as valuable mouse models for studying HSC biology.

In addition to validating the expression pattern of Hlf-tdTomato in FL and BM, which was principally consistent with the findings in a recent study (Yokomizo et al., 2019), we made an effort in the present study to explore the expression pattern of Hlf-tdTomato in early pre-HSCs with functional evaluation. Previous study has reported the presence of CD45^–^ pre-HSCs in the E10.5 AGM region (Rybtsov et al., 2011); however, whether there exist CD45^+^ pre-HSCs at E10.5 has been controversial (Boisset et al., 2015). Based on the *in vitro* co-culture system we used, the progenies of CD45^+^Hlf-tdTomato^+^ but not CD45^+^Hlf-tdTomato^–^ cells in the E10.5 AGM region were capable of repopulating irradiated adult recipients. Thus, we demonstrated the existence of CD45^+^ pre-HSCs in E10.5 AGM, which express Hlf. Moreover, our finding suggested that Hlf expression prior to CD45 expression is a prerequisite for the cells to keep the HSC competence. Unlike the CD45^+^ cells, CD45^–^ cells showed repopulating activity in both Hlf-tdTomato^+^ and Hlf-tdTomato^–^ counterparts. However, presently, we could not figure out to what extent the HSC-primed HEC population contributed to the repopulating capacity of the Hlf-tdTomato^–^ sub-population in the E10.5 AGM region. Further precise study designs are needed to address this question. It is conceivable that Hlf-tdTomato reporter can be used as an indicator of HSC emergence when carrying out reprogramming experiment or inducing ES cell differentiation *in vitro*.

Although a variety of mouse models have been used to trace the fate of HSCs, there is still room for improvement. Some mouse models have been used to mark adult HSCs, such as *Pdzk1ip1-CreERT2*, *Fgd5-CreERT2*, *Vwf-CreERT2*, *Krt18-CreERT2*, and *Tie2-MerCreMer*, with varied efficiencies to label adult HSCs when induced at adult stage (Busch et al., 2015; Sawai et al., 2016; Carrelha et al., 2018; Chapple et al., 2018; Sawen et al., 2018). However, the expression of most of these molecules is not specific to HSCs during development. Some mouse models, including *Cdh5-CreERT2*, *Tie2-MerCreMer*, and *Runx1-MerCreMer*, have been used to label embryonic HSCs when induced at mid-gestation (Zovein et al., 2008; Gomez Perdiguero et al., 2015; Hoeffel et al., 2015). Since these molecules are also expressed in the cells of other waves of hematopoiesis in addition to HSC generation during embryogenesis, and the occurring period for different waves of hematopoiesis overlaps to a certain extent, lineage tracing models using HSC-specific markers are urgently needed to more specifically and clearly delineate the fate of embryonic HSCs exclusively. Together with a previous study showing that Hlf is not expressed in erythro-myeloid progenitors, a population belonging to HSC-independent hematopoiesis (Yokomizo et al., 2019), our present findings suggested *Hlf-CreER* mice as an unprecedented model for studying embryonic HSC-derived hematopoiesis.

Our previous studies have reported the expression of CD201 (known as EPCR, encoded by *Procr*) in HSC-primed HECs and pre-HSCs, which is a stemness molecule and also expressed in aortic endothelial cells, as well as the contribution of CD201-labeled embryonic cells to fetal and adult blood lineages revealed by *Procr-CreER*-mediated lineage tracing when induced at E9.5 (Zhou et al., 2016; Lan, 2017; Zheng et al., 2019; Hou et al., 2020). Moreover, we have identified Neurl3 as a signature gene of HSC-primed HECs and pre-HSCs and achieved the enrichment of HSC-competent HECs by the *Neurl3-EGFP* reporter (Hou et al., 2020). In the future, by combining these mouse models, we will more precisely delineate the comprehensive roadmap for the diversified fate choice of different hematopoietic lineages from embryonic HSCs.

## Data Availability Statement

The original contributions presented in the study are included in the article/Supplementary Material, further inquiries can be directed to the corresponding author/s.

## Ethics Statement

The animal study was reviewed and approved by the Academy of Military Medical Sciences (Fifth Medical Center of Chinese PLA General Hospital).

## Author Contributions

YL and BL designed the study. WT performed the cell sorting, culture, immunostaining, and transplantation assays with help from JHe, TH, ZB, CW, HW, RY, YN, YY, YG, SH, JHo, JW, and JZ. WT, YL, and BL wrote the manuscript and revision. All authors reviewed the manuscript.

## Conflict of Interest

The authors declare that the research was conducted in the absence of any commercial or financial relationships that could be construed as a potential conflict of interest.

## Publisher’s Note

All claims expressed in this article are solely those of the authors and do not necessarily represent those of their affiliated organizations, or those of the publisher, the editors and the reviewers. Any product that may be evaluated in this article, or claim that may be made by its manufacturer, is not guaranteed or endorsed by the publisher.

## Supplementary Material

Supplementary Figure 1 Gating strategies for FACS analyses of Hlf-tdTomato expression in different hematopoietic populations from *Hlf-tdTomato* mice. **(A)** Representative FACS plots showing the expression of Hlf-tdTomato in Gr1^+^ cells and F4/80^+^ macrophages in E15.5 fetal liver (FL). Data are representative of two independent experiments. **(B)** Representative FACS plots showing Hlf-tdTomato expression in the indicated hematopoietic populations in the adult bone marrow (BM). LT-HSC, long-term hematopoietic stem cell; ST-HSC, short-term hematopoietic stem cell; MPP, multipotent progenitor; CMP, common myeloid progenitor; GMP, granulocyte-monocyte progenitor; MEP, megakaryocyte-erythroid progenitor. Data are representative of three independent experiments. **(C)** Representative FACS plots (left) and graph (right) showing the Hlf-tdTomato expression in LT-HSCs, ST-HSCs and MPPs in the bone marrow (BM) of adult *Hlf-tdTomato* mice. LSKFlk2CD34 marker combination was used. Data are from three independent experiments. **(D)** Representative FACS plots showing the Hlf-tdTomato expression in granulocytes/monocytes (GMs), B cells, and T cells in the peripheral blood of adult *Hlf-tdTomato* mice. Data are representative of two independent experiments.

Supplementary Figure 2 Representative FACS analyses of Hlf-tdTomato expression in embryos. **(A)** Representative FACS plots showing the proportions of Hlf-tdTomato^+^ cells in hematopoietic cells (CD41^+^/CD43^+^/CD45^+^) and non-hematopoietic cells (CD41^–^CD43^–^CD45^–^) of the limb, head, and trunk from E10.5 *Hlf-tdTomato* embryos. Data are representative of two independent experiments. **(B)** Representative FACS plots showing no Hlf-tdTomato expression in PK44 (CD41^–^CD43^–^CD45^–^CD31^+^CD44^+^Kit^+^CD201^+^) population of the E10.5 *Hlf-tdTomato* AGM region.

Supplementary Figure 3 Representative FACS analyses of multi-lineage repopulation in multiple organs. **(A)** Representative FACS plots showing multi-organ and multi-lineage repopulation in the recipients 16 weeks post-transplantation transplanted with the culture products of the indicated cell populations from the E10.5 AGM region. The myeloid cells (Gr1/Mac-1^+^), B cells (B220^+^), and T cells (CD3^+^ or CD4/CD8^+^) in multiple hematopoietic organs are shown. **(B)** Representative FACS plots showing the proportions of donor-derived cells (represented by CD45.1^+^) in hematopoietic stem progenitor populations in the bone marrow of the reconstituted recipients 16 weeks post-transplantation transplanted with the progenies of the indicated cell populations from the E10.5 AGM region. LSKSLAM marker combination was used. **(C)** Representative FACS plots showing multi-organ and multi-lineage repopulation in the recipient 16 weeks post-transplantation transplanted with the progenies of the indicated cell populations from the E10.5 AGM region. The myeloid cells (Gr1/Mac-1^+^), B cells (B220^+^), and T cells (CD3^+^ or CD4/CD8^+^) in multiple hematopoietic organs are shown. **(D)** Representative FACS plots showing the proportions of donor-derived cells (represented by CD45.1^+^) in hematopoietic stem progenitor populations in the bone marrow of the reconstituted recipient 16 weeks post-transplantation transplanted with the progenies of the indicated cell populations from the E10.5 AGM region. LSKSLAM marker combination was used. LT-HSC, long-term hematopoietic stem cell; ST-HSC, short-term hematopoietic stem cell; MPP, multipotent progenitor; CMP, common myeloid progenitor; GMP, granulocyte-monocyte progenitor; MEP, megakaryocyte-erythroid progenitor.

Supplementary Figure 4 Representative FACS analyses of lineage-traced cells in *Hlf-CreER;ZsGreen* mice. **(A)** Representative FACS plots showing the Hlf-labeled cells in endothelial cells (ECs), IAHCs, and T1 and T2 pre-HSCs in the AGM region of E11.5 *Hlf-CreER;ZsGreen* embryos with tamoxifen administrated at E9.5. Data are representative of six independent experiments. **(B)** Graph showing the dynamics of labeling in endothelial cells (ECs), IAHCs, and T1 and T2 pre-HSCs in the AGM region of E11.5 *Hlf-CreER;ZsGreen* embryos with tamoxifen administration at E9.5. Each line represents an individual embryo. **(C)** Representative FACS plots (left) and graph (right) showing the proportions of Hlf-labeled cells in the immunophenotypic HSCs (ESLAM) in the fetal liver (FL) of E15.5 *Hlf-CreER;ZsGreen* embryos with tamoxifen administration at E9.5. Data are representative of four independent experiments.
