# Supplementary figures and images for: Hlf Expression Marks Early Emergence of Hematopoietic Stem Cell Precursors With Adult Repopulating Potential and Fate
Source: Front Cell Dev Biol. 2021 Sep 13;9:728057. doi: 10.3389/fcell.2021.728057 (PMC8473784; doi:10.3389/fcell.2021.728057)

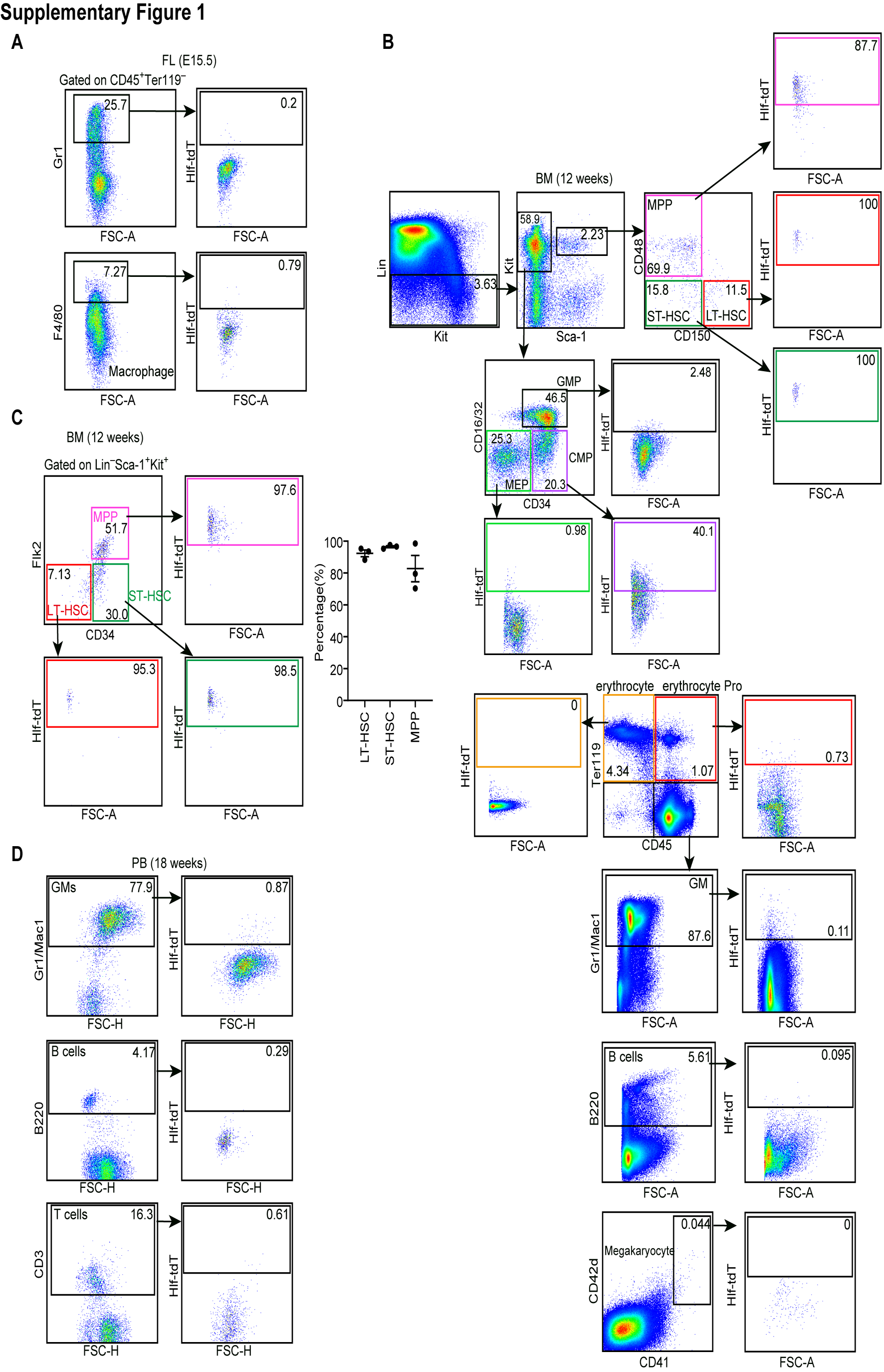

Supplement: Supplementary Figure 1 — Gating strategies for FACS analyses of Hlf-tdTomato expression in different hematopoietic populations from Hlf-tdTomato mice. (A) Representative FACS plots showing the expression of Hlf-tdTomato in Gr1+ cells and F4/80+ macrophages in E15.5 fetal liver (FL). Data are representative of two independent experiments. (B) Representative FACS plots showing Hlf-tdTomato expression in the indicated hematopoietic populations in the adult bone marrow (BM). LT-HSC, long-term hematopoietic stem cell; ST-HSC, short-term hematopoietic stem cell; MPP, multipotent progenitor; CMP, common myeloid progenitor; GMP, granulocyte-monocyte progenitor; MEP, megakaryocyte-erythroid progenitor. Data are representative of three independent experiments. (C) Representative FACS plots (left) and graph (right) showing the Hlf-tdTomato expression in LT-HSCs, ST-HSCs and MPPs in the bone marrow (BM) of adult Hlf-tdTomato mice. LSKFlk2CD34 marker combination was used. Data are from three independent experiments. (D) Representative FACS plots showing the Hlf-tdTomato expression in granulocytes/monocytes (GMs), B cells, and T cells in the peripheral blood of adult Hlf-tdTomato mice. Data are representative of two independent experiments. [file Image_1.TIF]

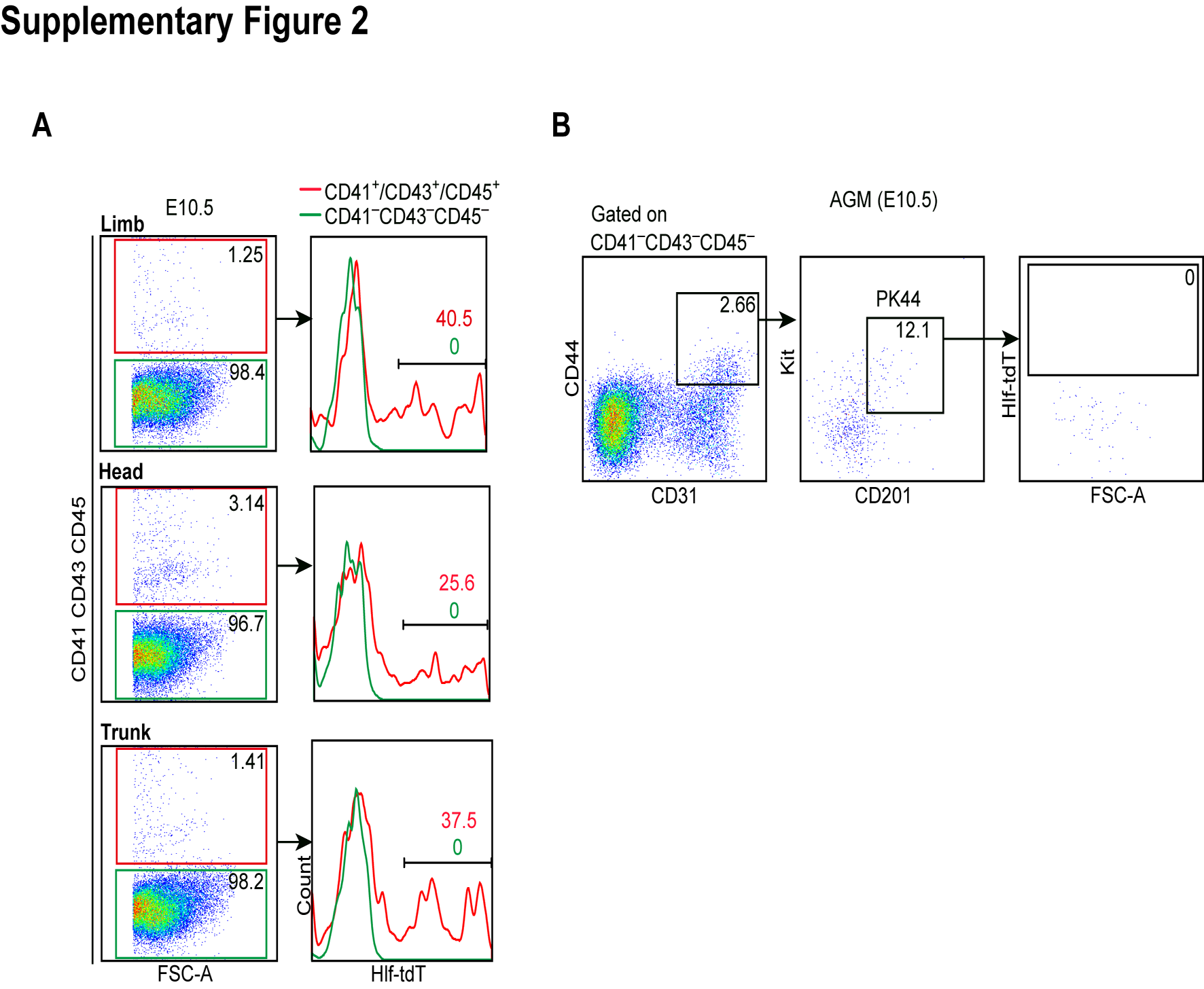

Supplement: Supplementary Figure 2 — Representative FACS analyses of Hlf-tdTomato expression in embryos. (A) Representative FACS plots showing the proportions of Hlf-tdTomato+ cells in hematopoietic cells (CD41+/CD43+/CD45+) and non-hematopoietic cells (CD41–CD43–CD45–) of the limb, head, and trunk from E10.5 Hlf-tdTomato embryos. Data are representative of two independent experiments. (B) Representative FACS plots showing no Hlf-tdTomato expression in PK44 (CD41–CD43–CD45–CD31+CD44+Kit+CD201+) population of the E10.5 Hlf-tdTomato AGM region. [file Image_2.TIF]

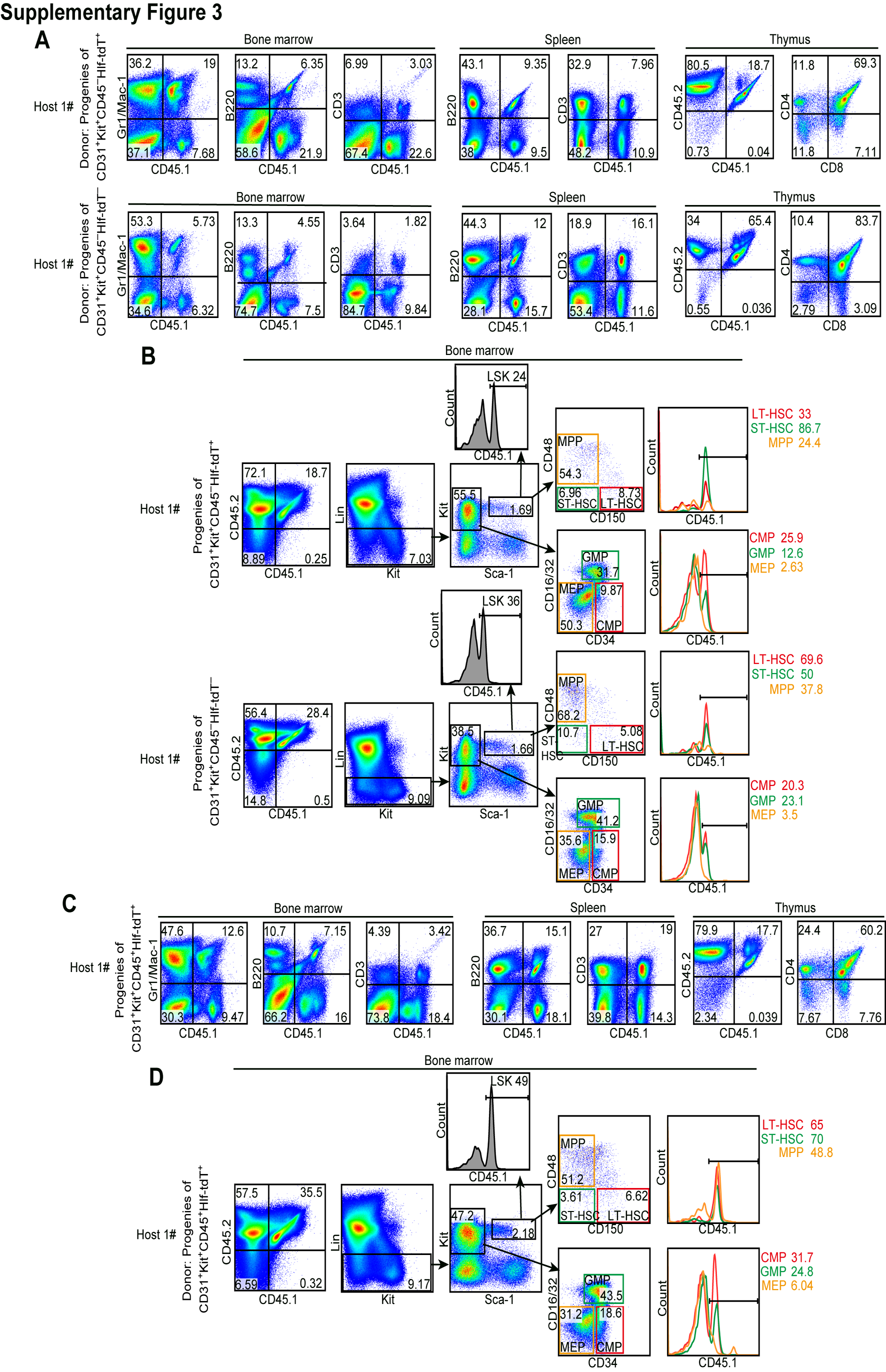

Supplement: Supplementary Figure 3 — Representative FACS analyses of multi-lineage repopulation in multiple organs. (A) Representative FACS plots showing multi-organ and multi-lineage repopulation in the recipients 16 weeks post-transplantation transplanted with the culture products of the indicated cell populations from the E10.5 AGM region. The myeloid cells (Gr1/Mac-1+), B cells (B220+), and T cells (CD3+ or CD4/CD8+) in multiple hematopoietic organs are shown. (B) Representative FACS plots showing the proportions of donor-derived cells (represented by CD45.1+) in hematopoietic stem progenitor populations in the bone marrow of the reconstituted recipients 16 weeks post-transplantation transplanted with the progenies of the indicated cell populations from the E10.5 AGM region. LSKSLAM marker combination was used. (C) Representative FACS plots showing multi-organ and multi-lineage repopulation in the recipient 16 weeks post-transplantation transplanted with the progenies of the indicated cell populations from the E10.5 AGM region. The myeloid cells (Gr1/Mac-1+), B cells (B220+), and T cells (CD3+ or CD4/CD8+) in multiple hematopoietic organs are shown. (D) Representative FACS plots showing the proportions of donor-derived cells (represented by CD45.1+) in hematopoietic stem progenitor populations in the bone marrow of the reconstituted recipient 16 weeks post-transplantation transplanted with the progenies of the indicated cell populations from the E10.5 AGM region. LSKSLAM marker combination was used. LT-HSC, long-term hematopoietic stem cell; ST-HSC, short-term hematopoietic stem cell; MPP, multipotent progenitor; CMP, common myeloid progenitor; GMP, granulocyte-monocyte progenitor; MEP, megakaryocyte-erythroid progenitor. [file Image_3.TIF]

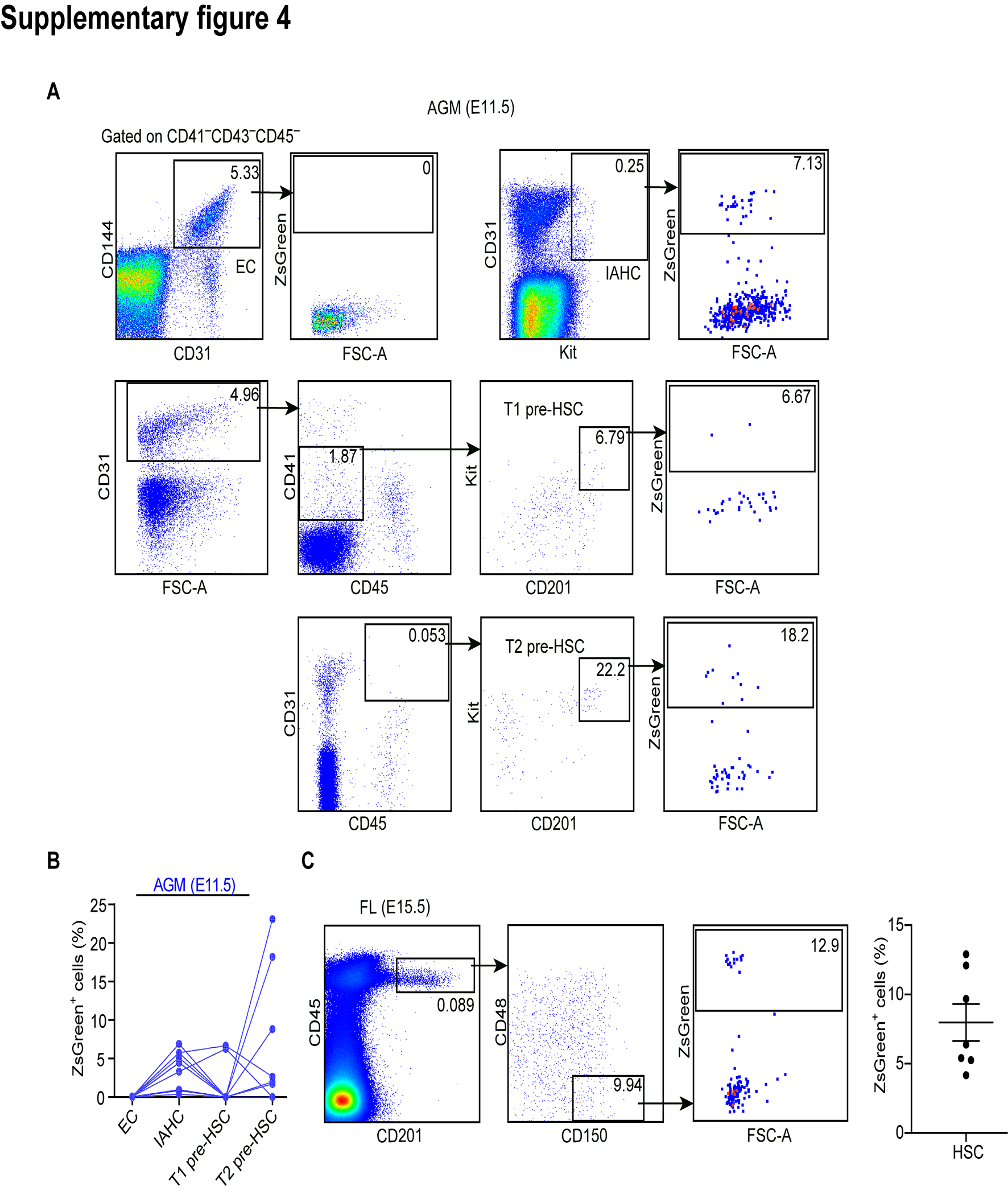

Supplement: Supplementary Figure 4 — Representative FACS analyses of lineage-traced cells in Hlf-CreER;ZsGreen mice. (A) Representative FACS plots showing the Hlf-labeled cells in endothelial cells (ECs), IAHCs, and T1 and T2 pre-HSCs in the AGM region of E11.5 Hlf-CreER;ZsGreen embryos with tamoxifen administrated at E9.5. Data are representative of six independent experiments. (B) Graph showing the dynamics of labeling in endothelial cells (ECs), IAHCs, and T1 and T2 pre-HSCs in the AGM region of E11.5 Hlf-CreER;ZsGreen embryos with tamoxifen administration at E9.5. Each line represents an individual embryo. (C) Representative FACS plots (left) and graph (right) showing the proportions of Hlf-labeled cells in the immunophenotypic HSCs (ESLAM) in the fetal liver (FL) of E15.5 Hlf-CreER;ZsGreen embryos with tamoxifen administration at E9.5. Data are representative of four independent experiments. [file Image_4.TIF]

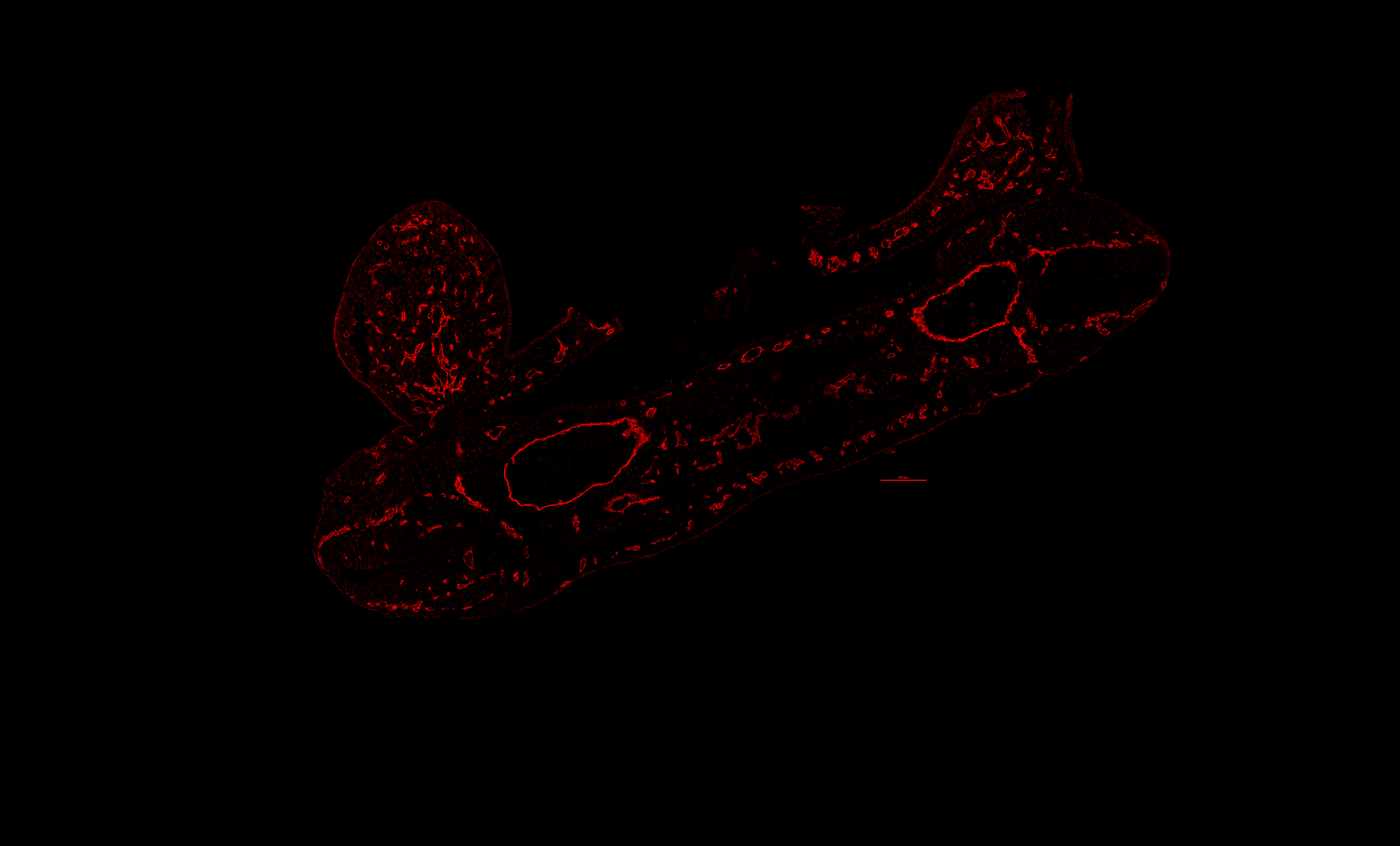

Supplement: Supplementary file 5 [file Data_Sheet_1.ZIP › Image1/Figure 1E/AGM_RGB_Cy5.tif]

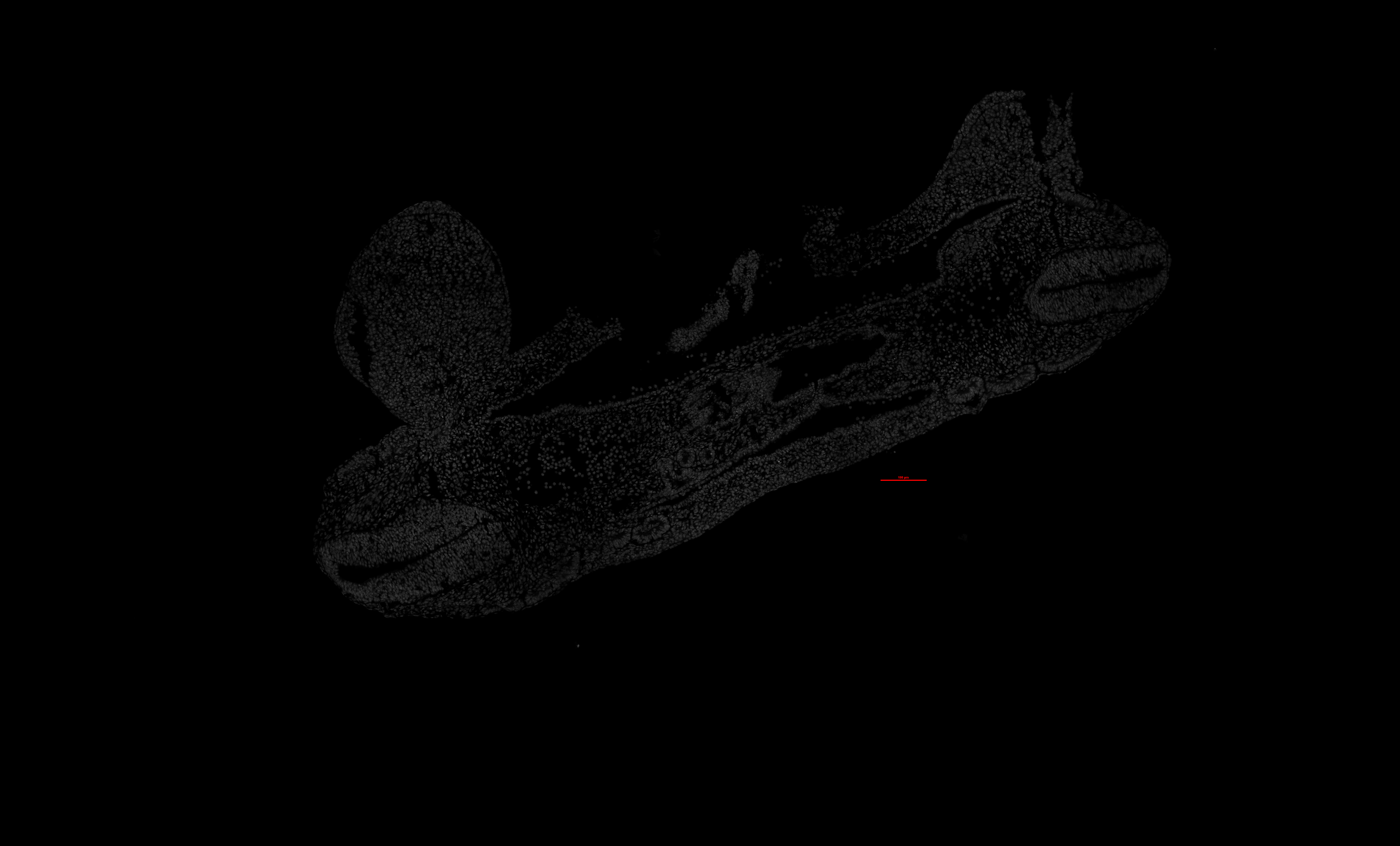

Supplement: Supplementary file 5 [file Data_Sheet_1.ZIP › Image1/Figure 1E/AGM-1_RGB_DAPI.tif]

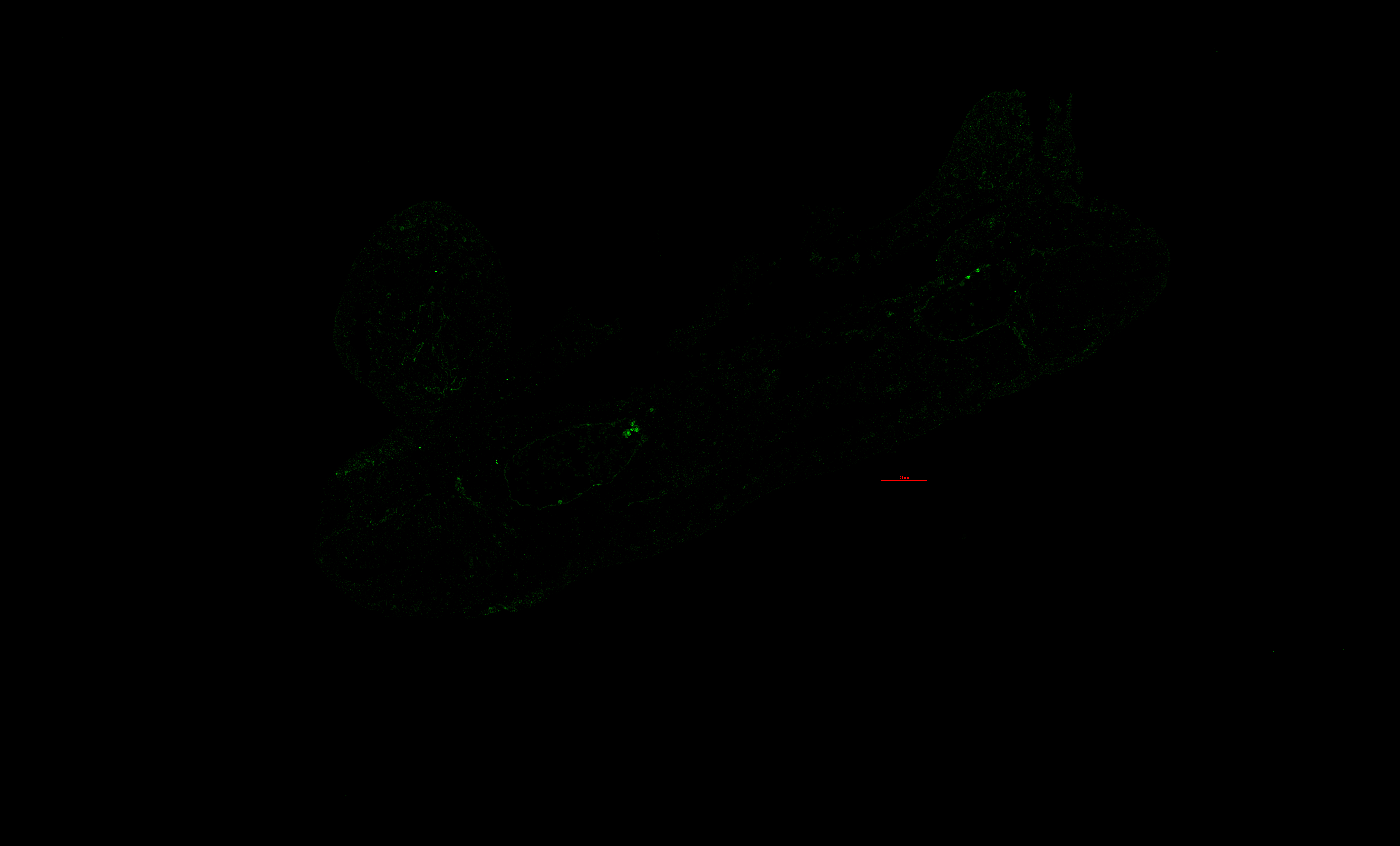

Supplement: Supplementary file 5 [file Data_Sheet_1.ZIP › Image1/Figure 1E/AGM-1_RGB_FITC.tif]

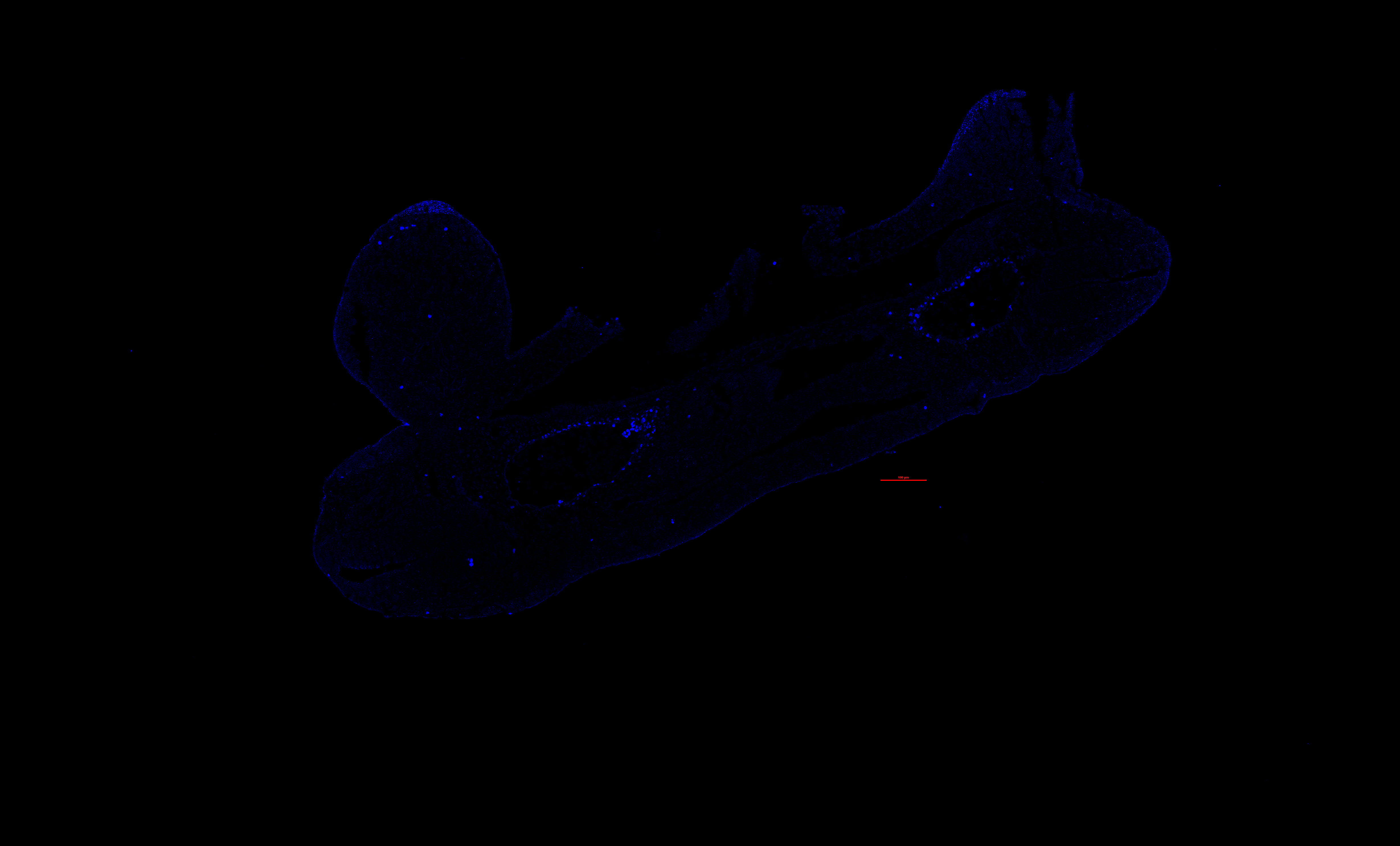

Supplement: Supplementary file 5 [file Data_Sheet_1.ZIP › Image1/Figure 1E/AGM-1_RGB_TRITC.tif]

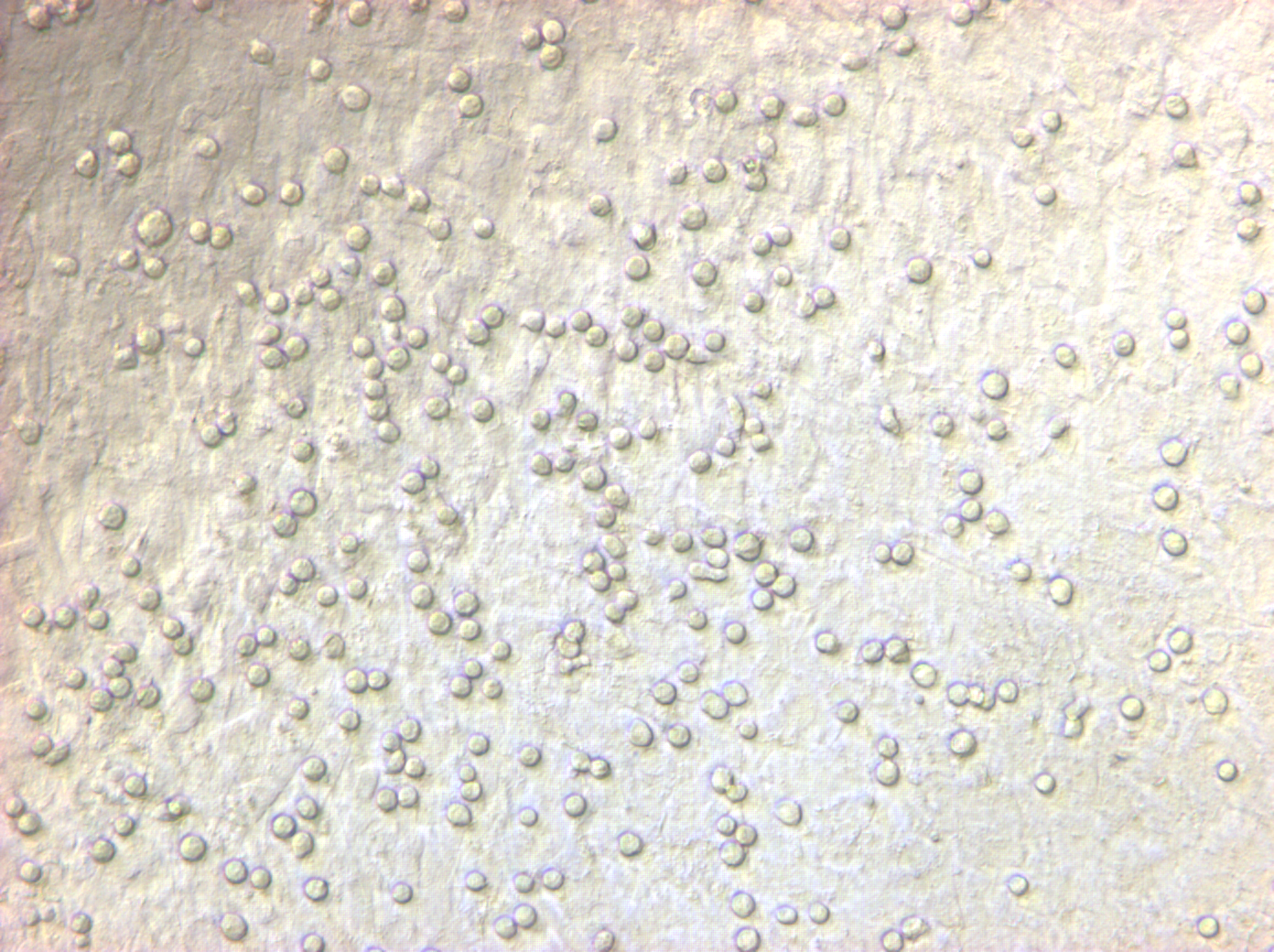

Supplement: Supplementary file 5 [file Data_Sheet_1.ZIP › Image1/Figure 2B/CD45-Hlf-.tif]

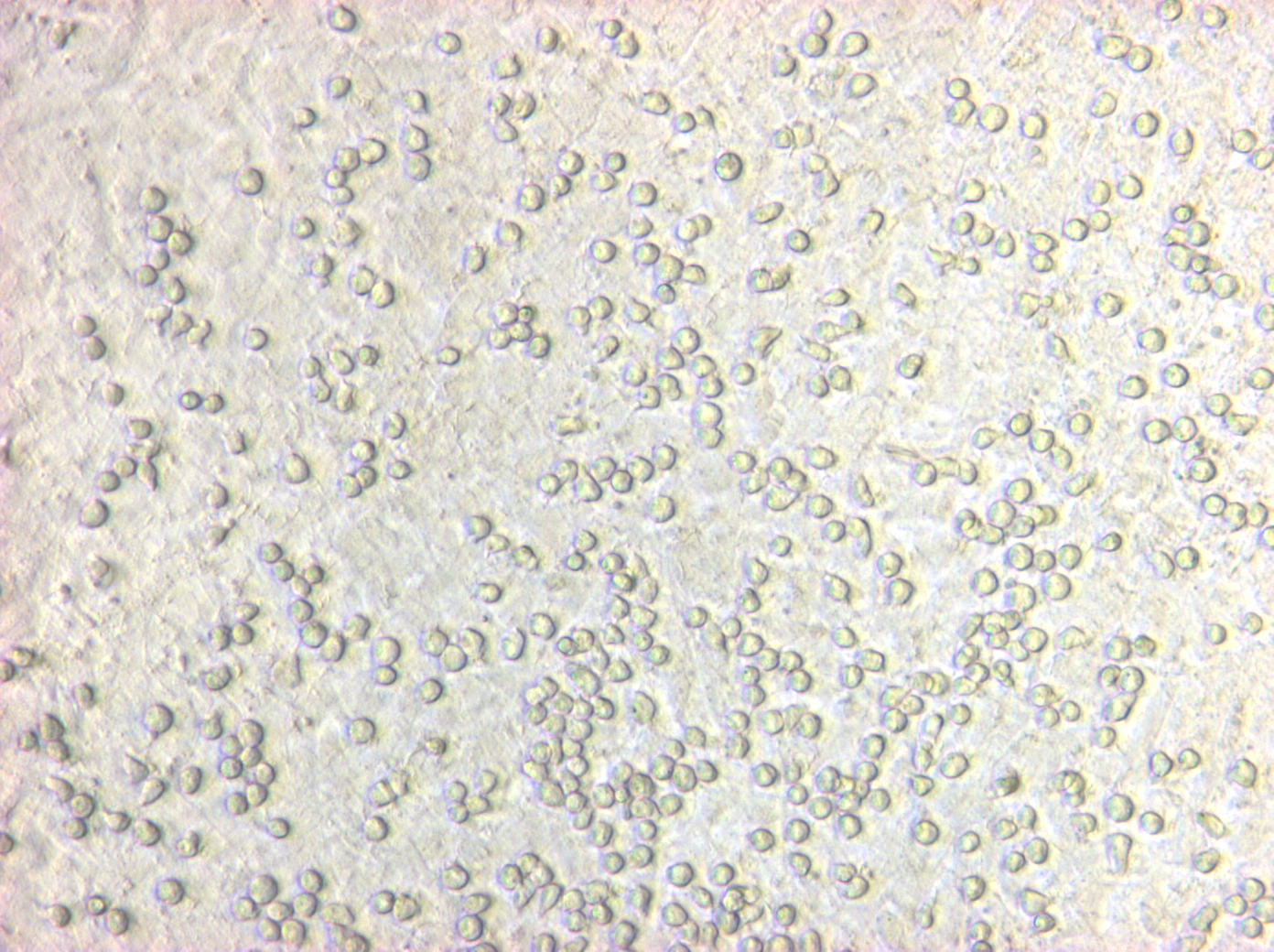

Supplement: Supplementary file 5 [file Data_Sheet_1.ZIP › Image1/Figure 2B/CD45-Hlf+.tif]

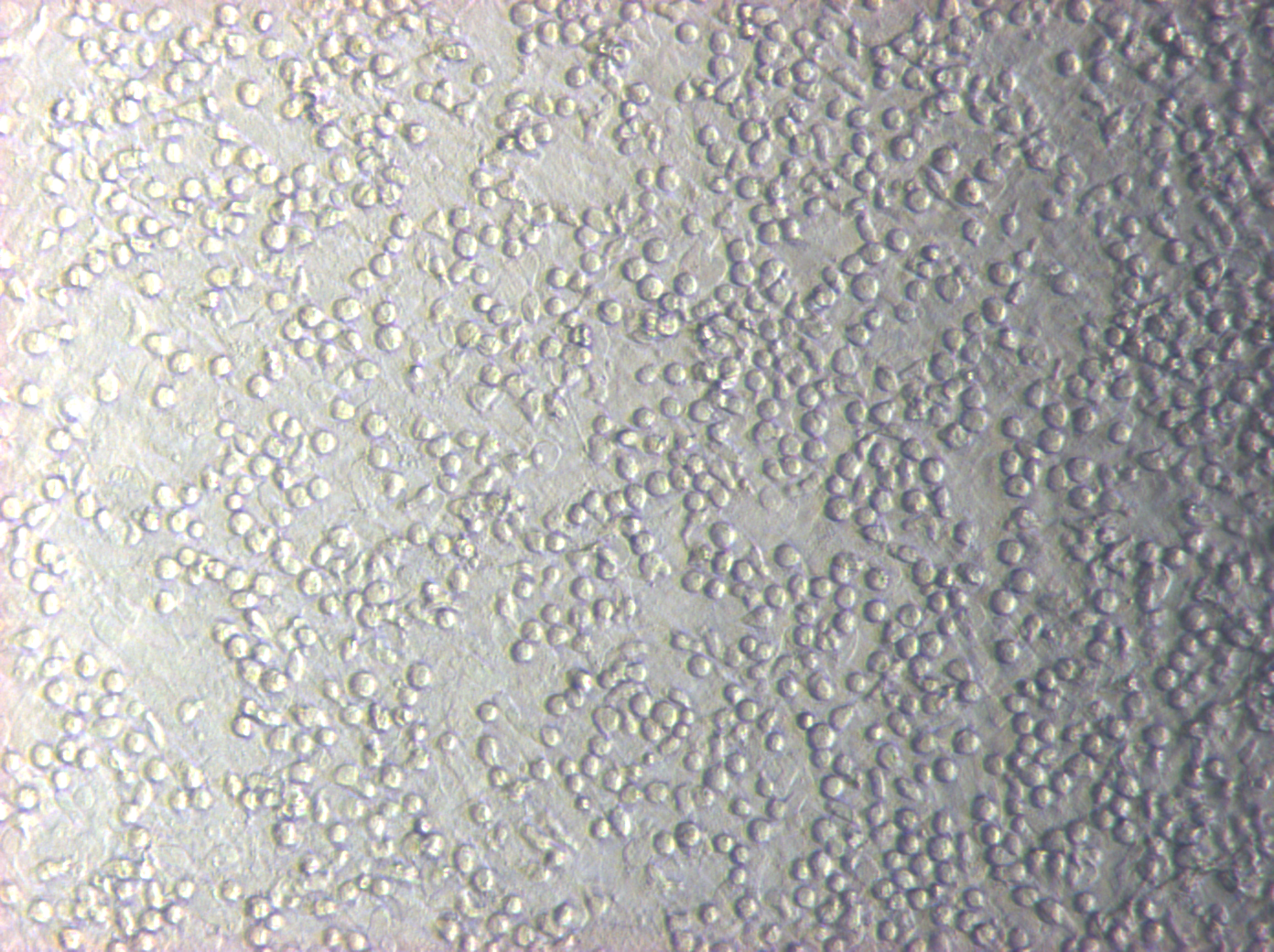

Supplement: Supplementary file 6 [file Data_Sheet_2.ZIP › Image2/Figure 3C/CD45+Hlf-.tif]

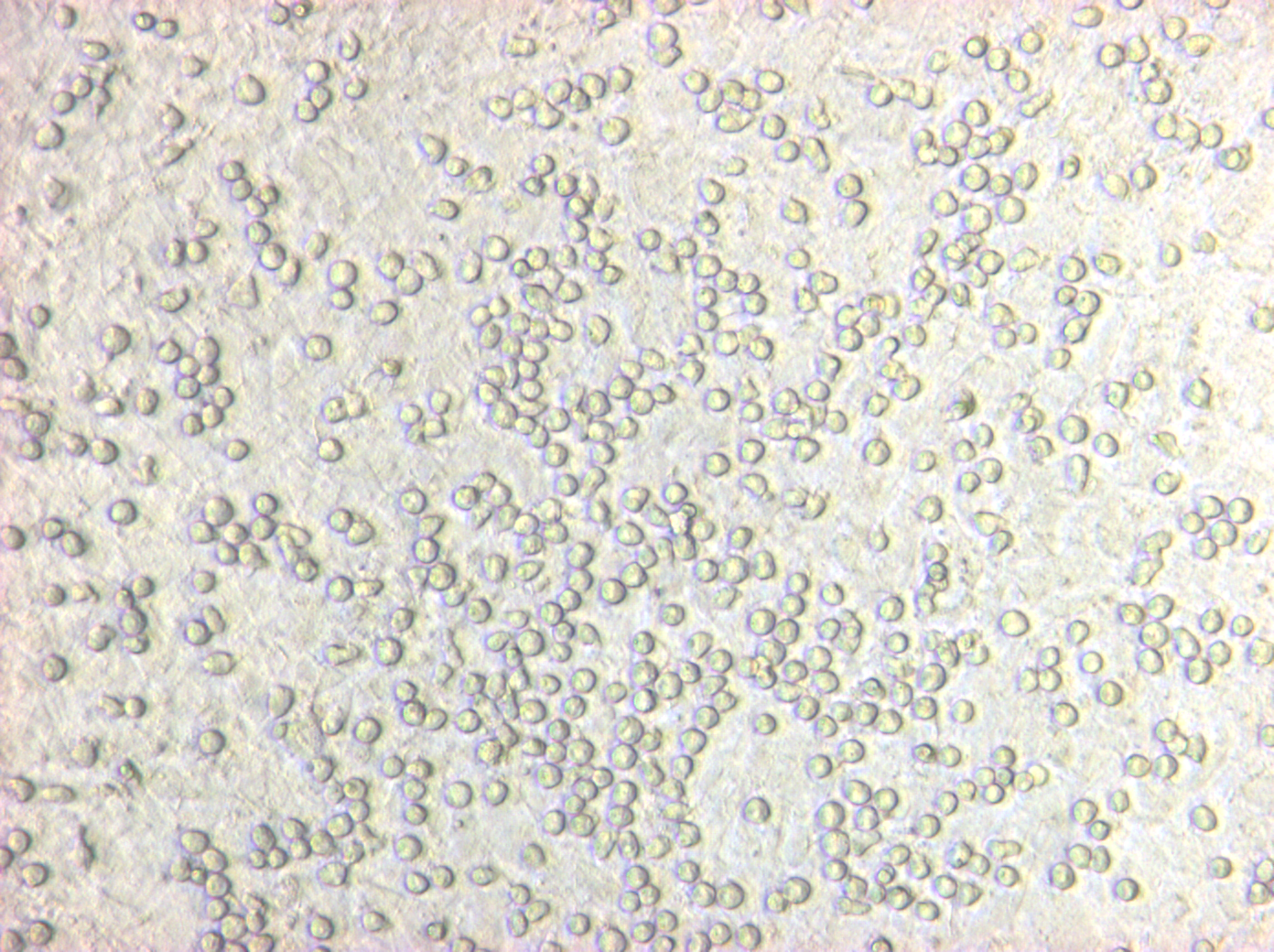

Supplement: Supplementary file 6 [file Data_Sheet_2.ZIP › Image2/Figure 3C/CD45+HlF+.tif]

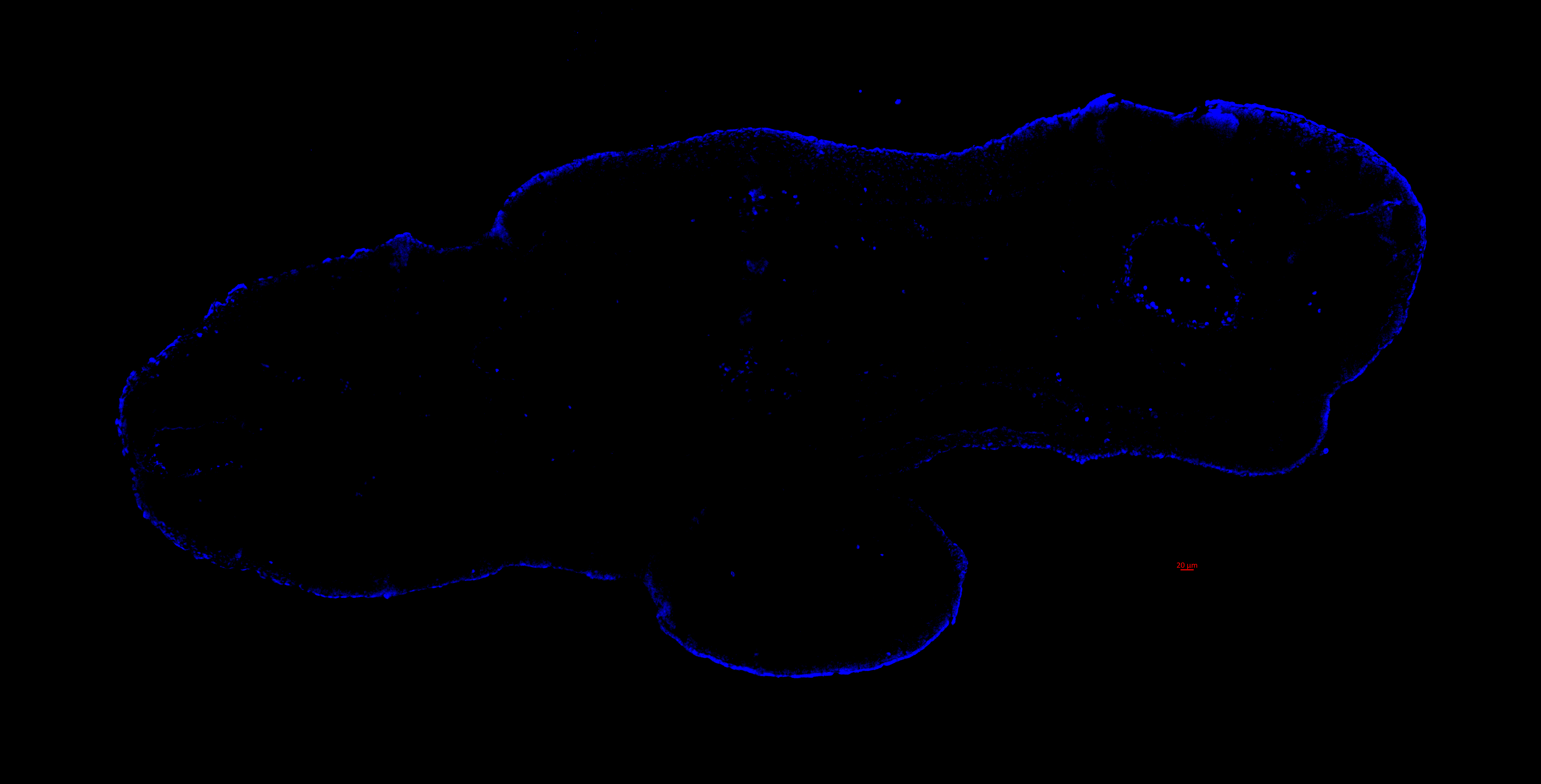

Supplement: Supplementary file 6 [file Data_Sheet_2.ZIP › Image2/Figure 4B/E10.0/sp32_c1.tif]

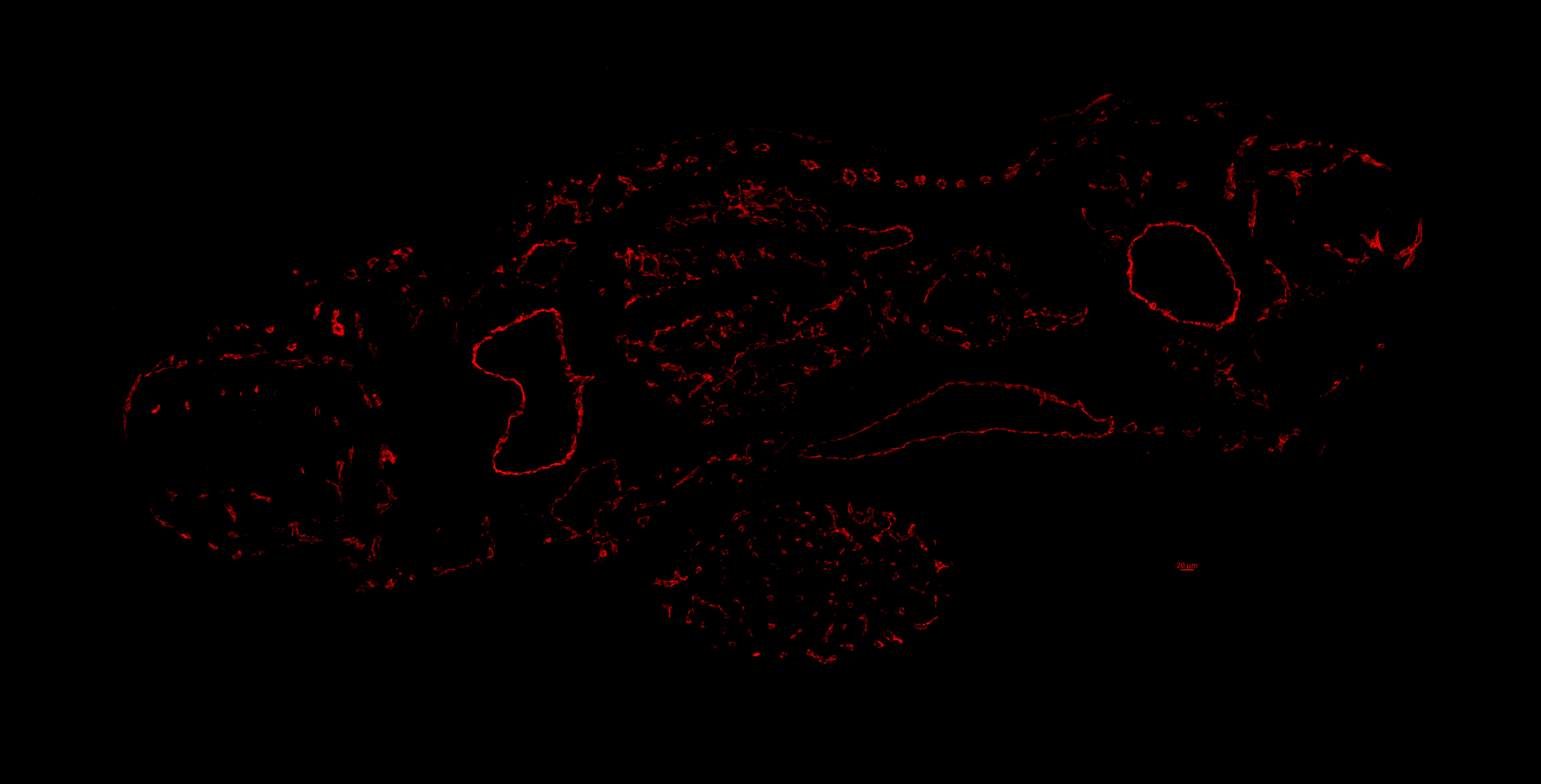

Supplement: Supplementary file 6 [file Data_Sheet_2.ZIP › Image2/Figure 4B/E10.0/sp32_c2.tif]

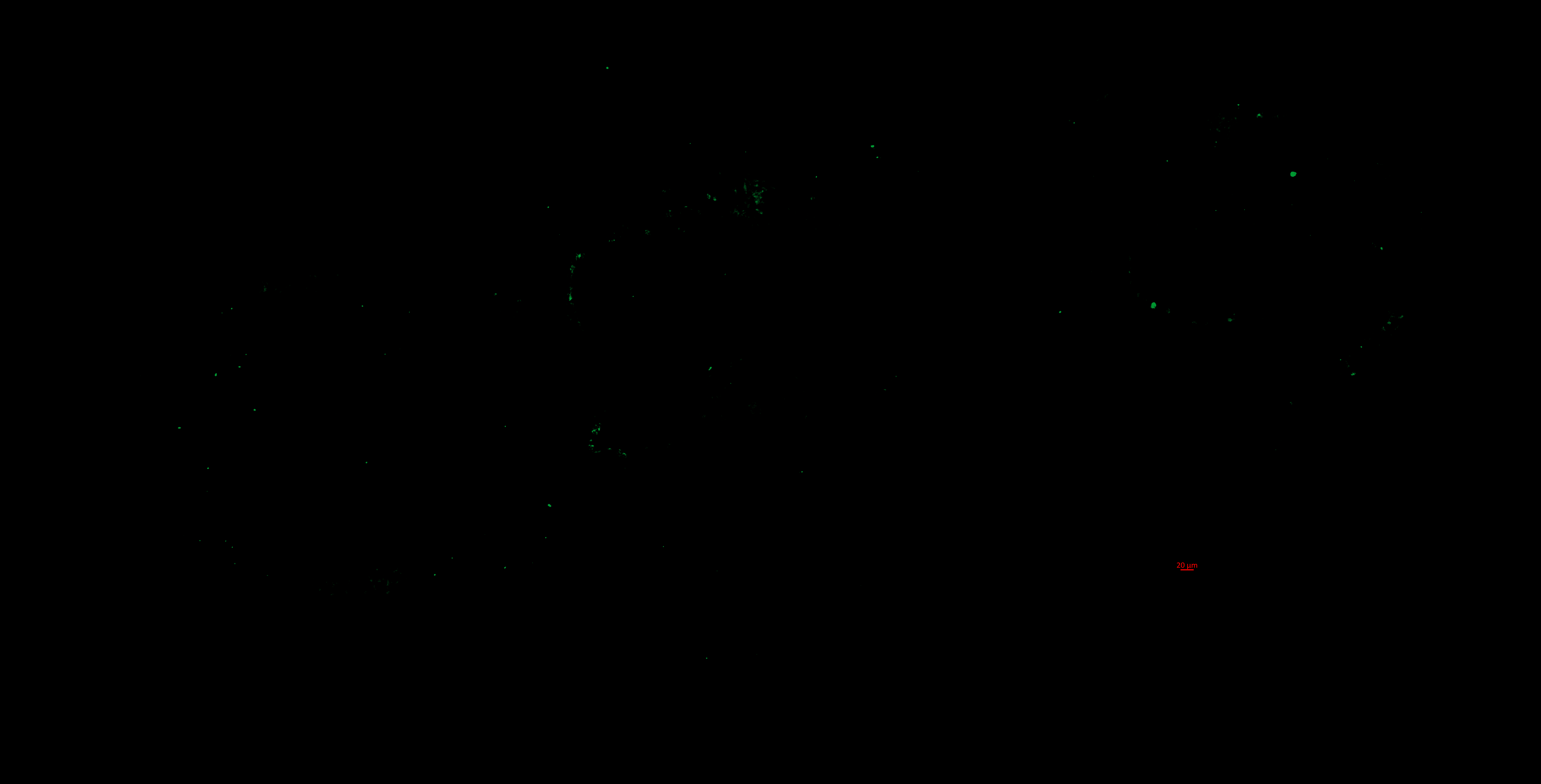

Supplement: Supplementary file 6 [file Data_Sheet_2.ZIP › Image2/Figure 4B/E10.0/sp32_c3.tif]

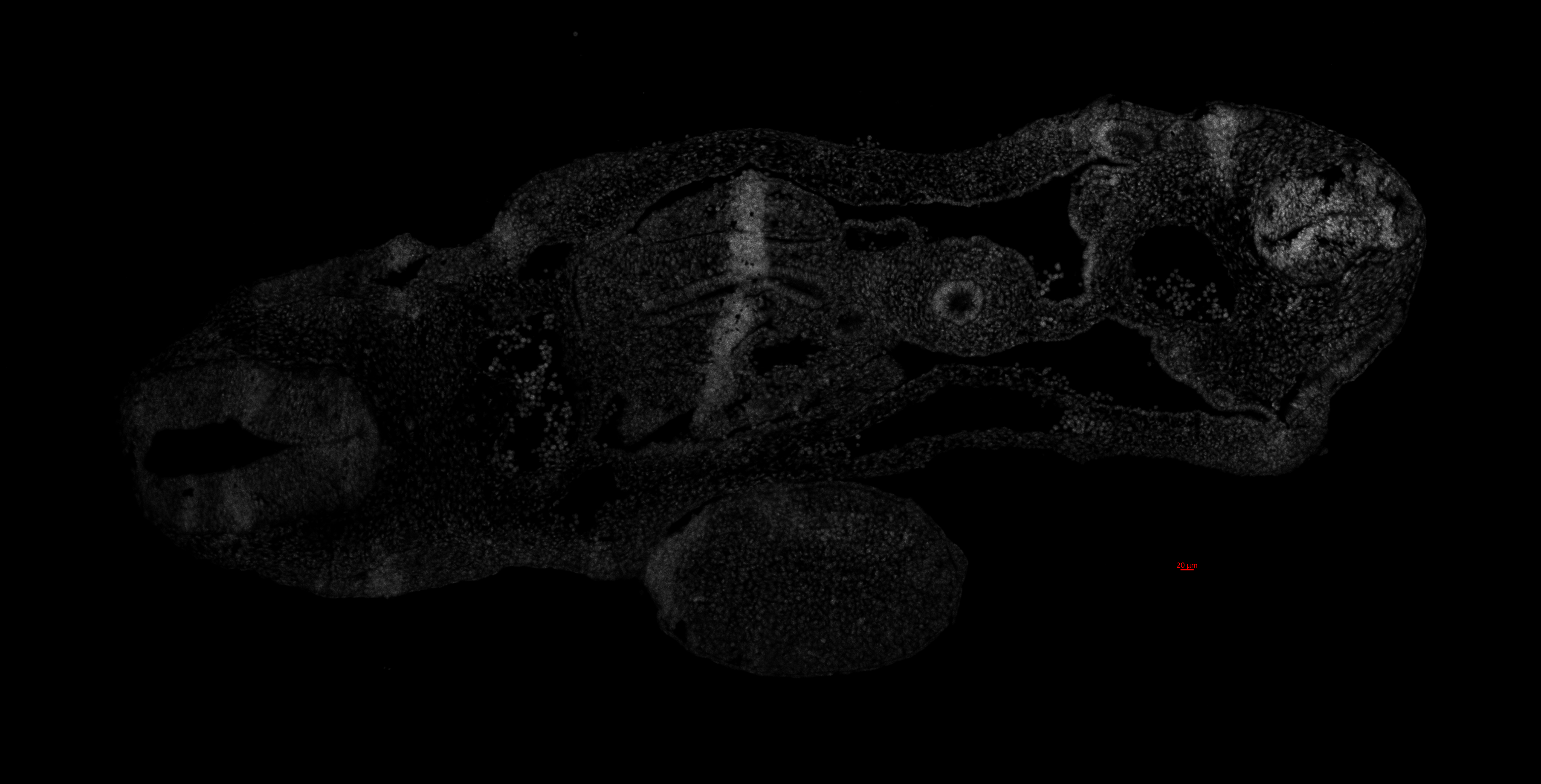

Supplement: Supplementary file 6 [file Data_Sheet_2.ZIP › Image2/Figure 4B/E10.0/sp32_c4.tif]

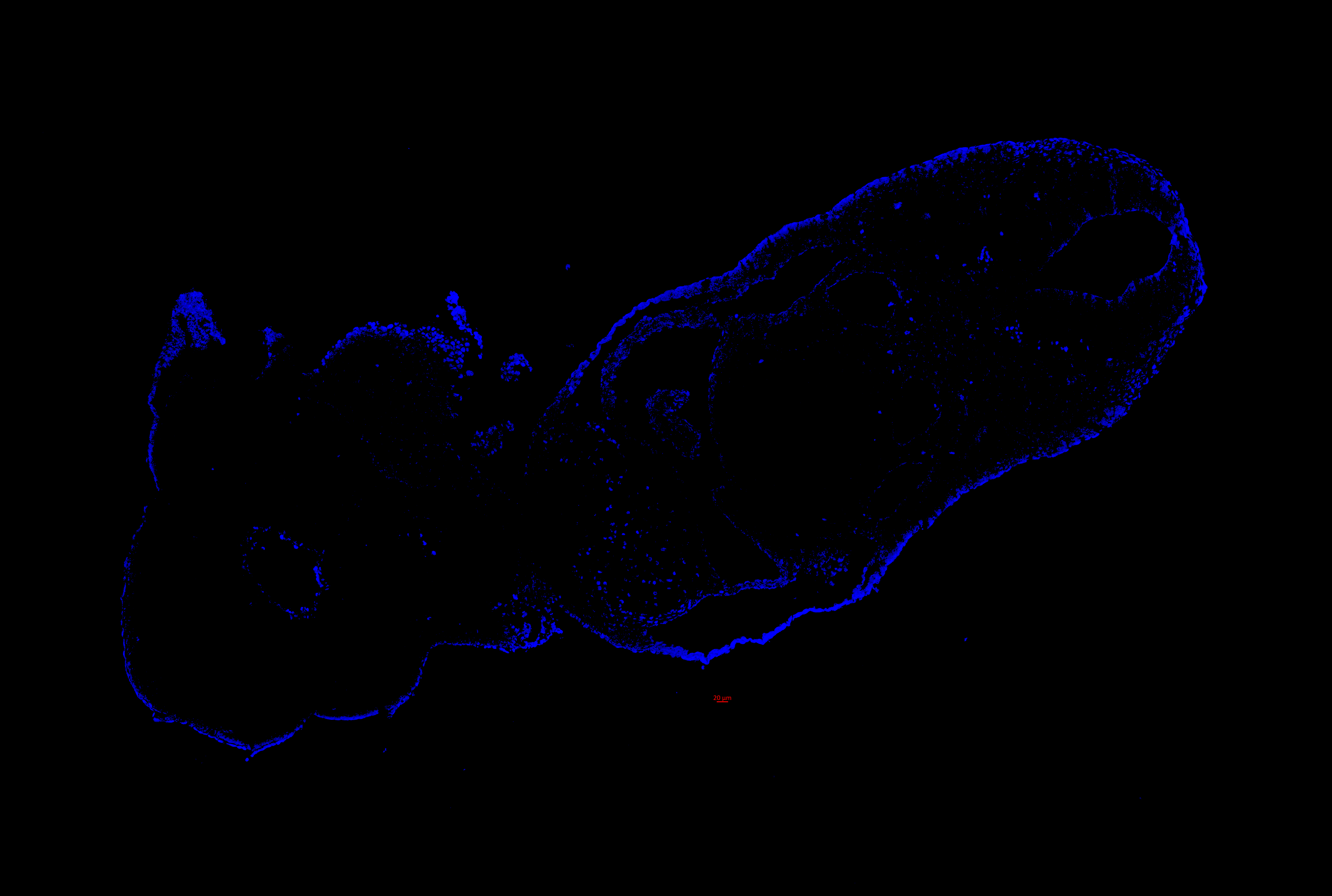

Supplement: Supplementary file 6 [file Data_Sheet_2.ZIP › Image2/Figure 4B/E10.5/sp38-20x_c1.tif]

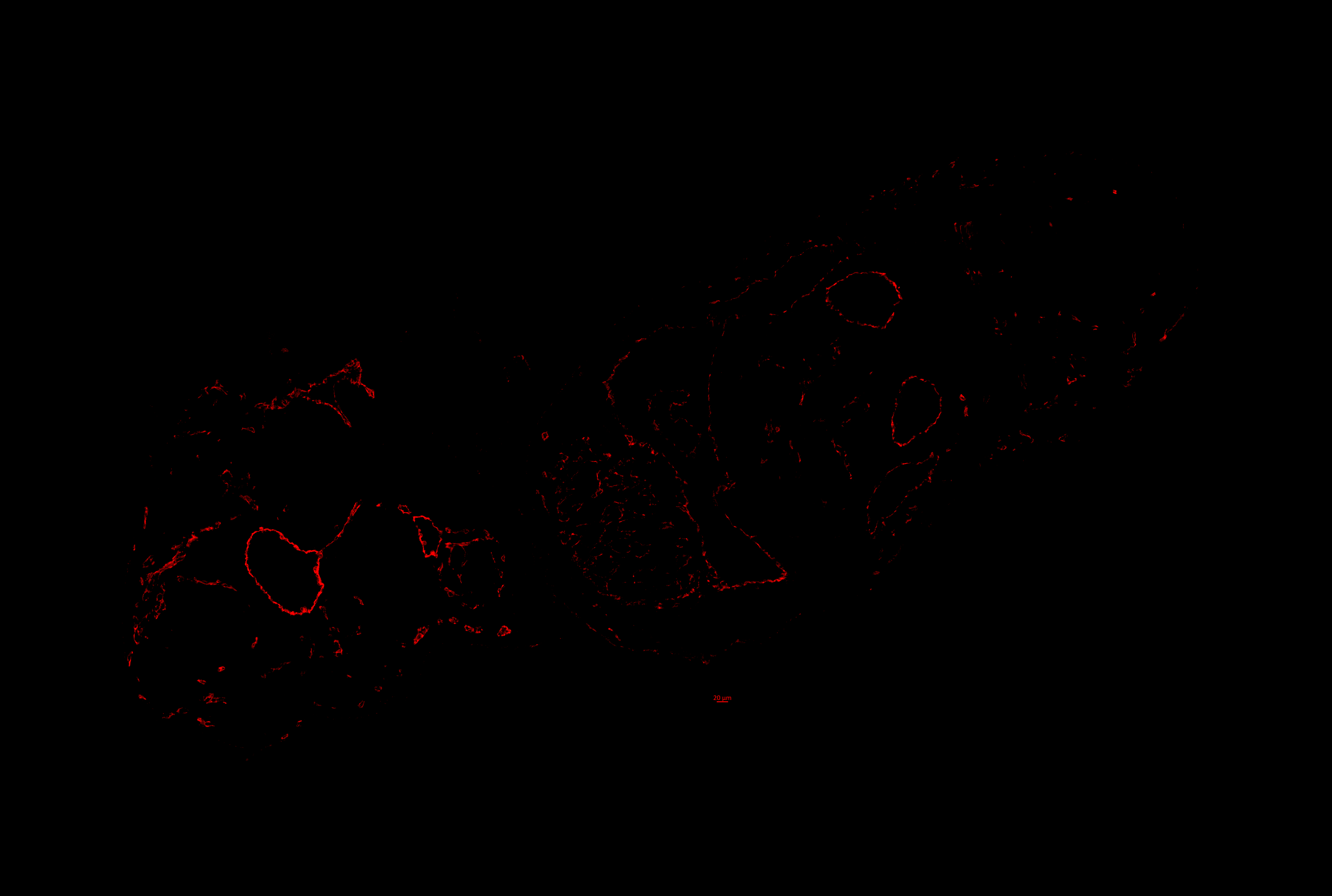

Supplement: Supplementary file 6 [file Data_Sheet_2.ZIP › Image2/Figure 4B/E10.5/sp38-20x_c2.tif]

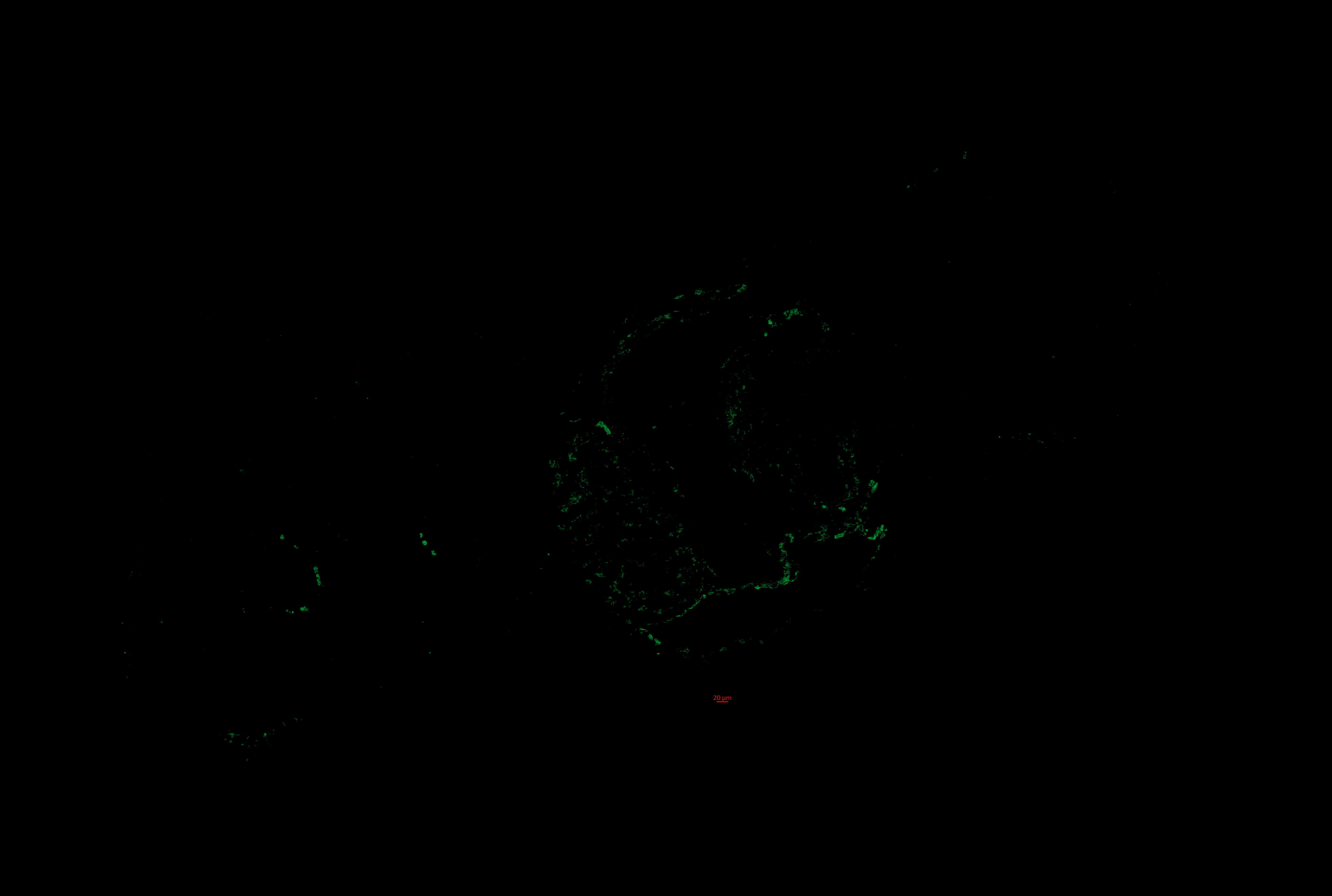

Supplement: Supplementary file 6 [file Data_Sheet_2.ZIP › Image2/Figure 4B/E10.5/sp38-20x_c3.tif]

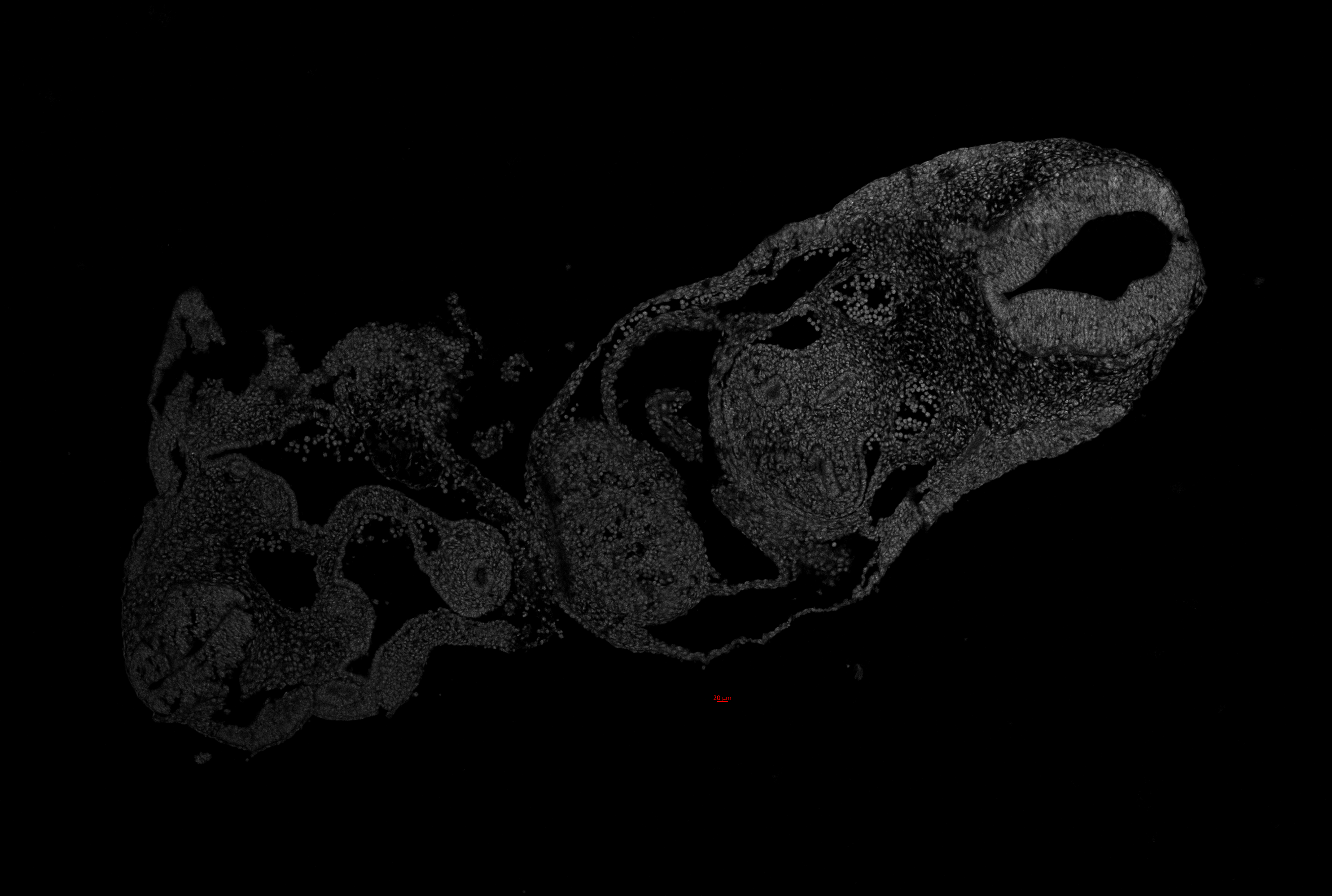

Supplement: Supplementary file 6 [file Data_Sheet_2.ZIP › Image2/Figure 4B/E10.5/sp38-20x_c4.tif]

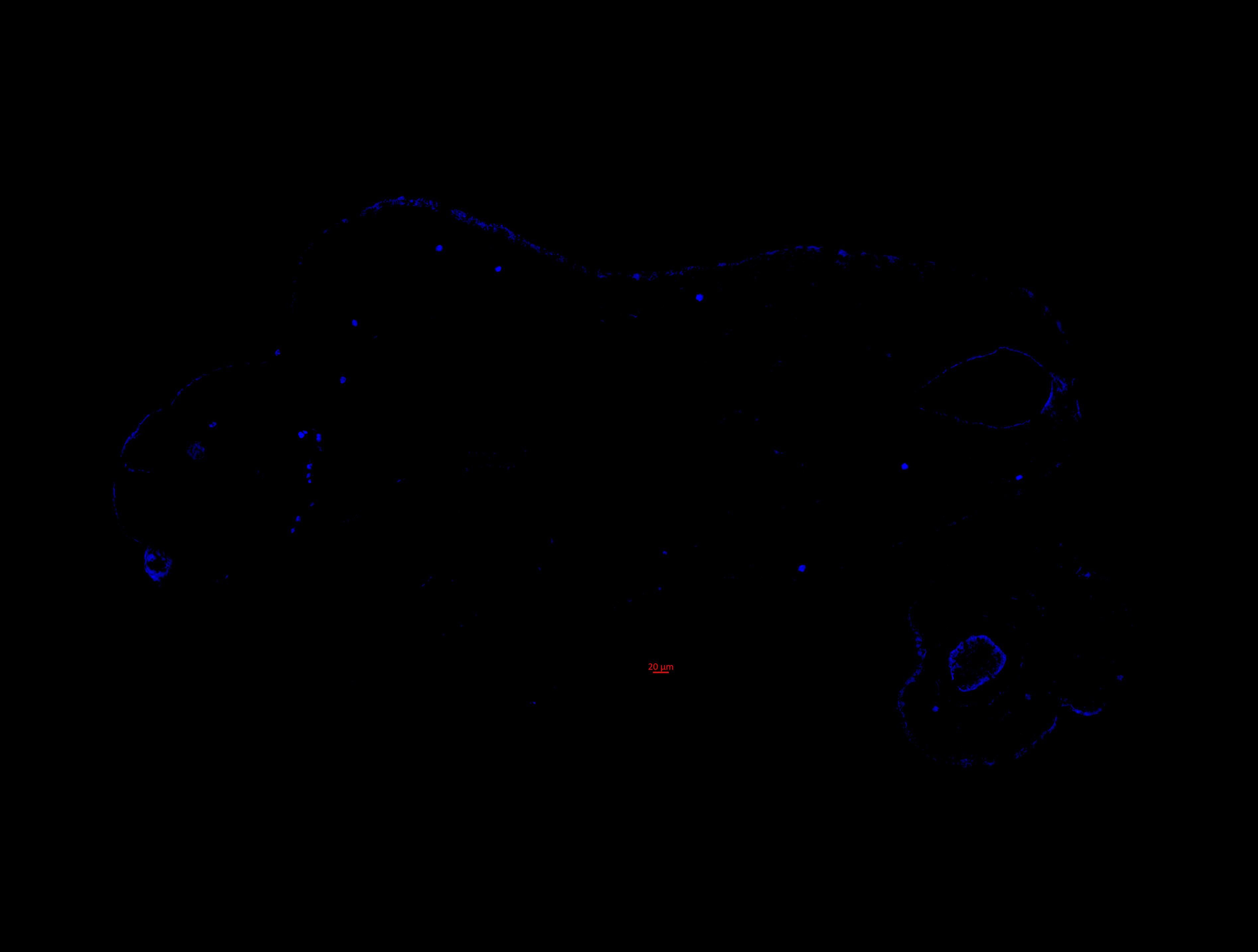

Supplement: Supplementary file 6 [file Data_Sheet_2.ZIP › Image2/Figure 4B/E9.5/sp27-9-AGM-20x_c1.tif]

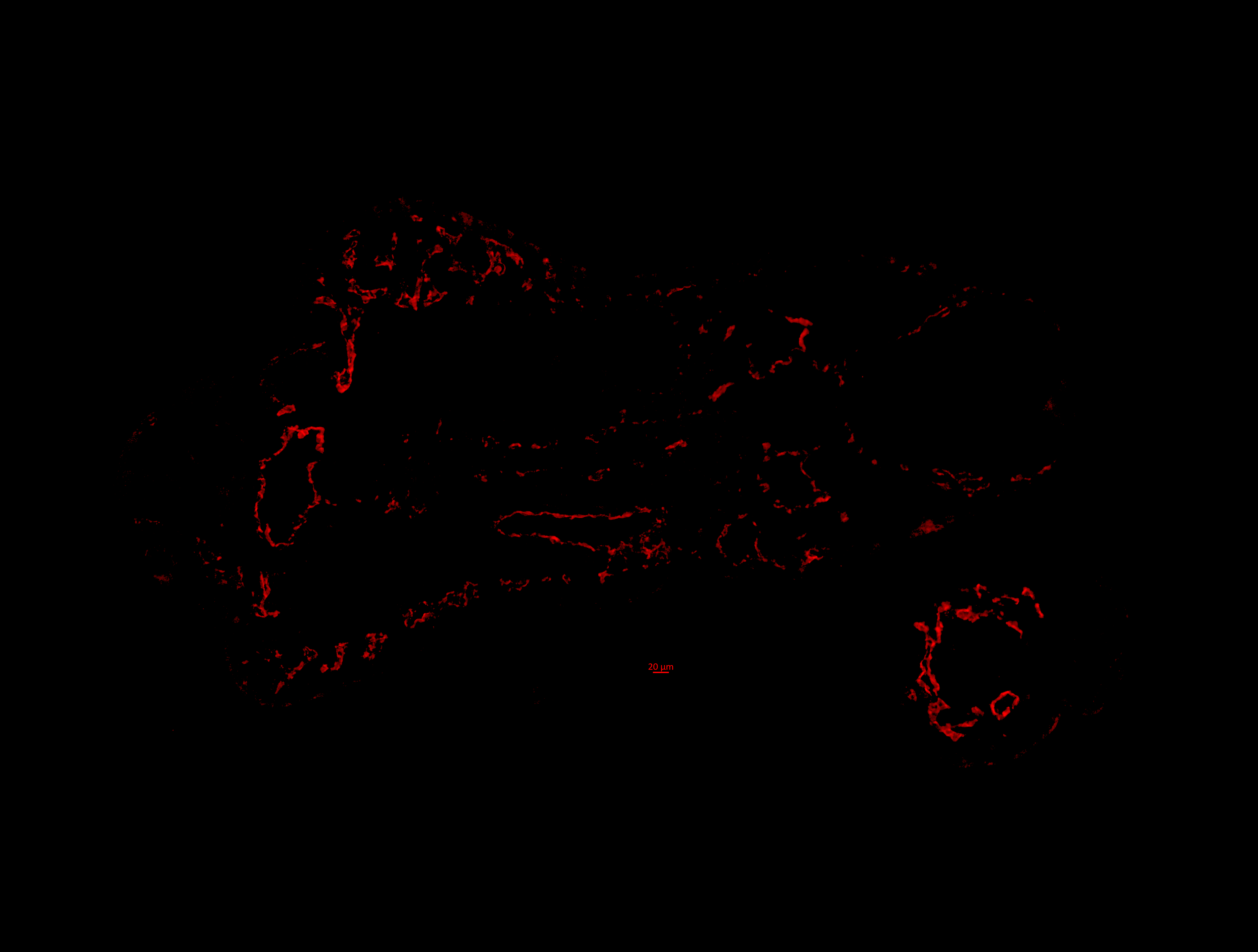

Supplement: Supplementary file 6 [file Data_Sheet_2.ZIP › Image2/Figure 4B/E9.5/sp27-9-AGM-20x_c2.tif]

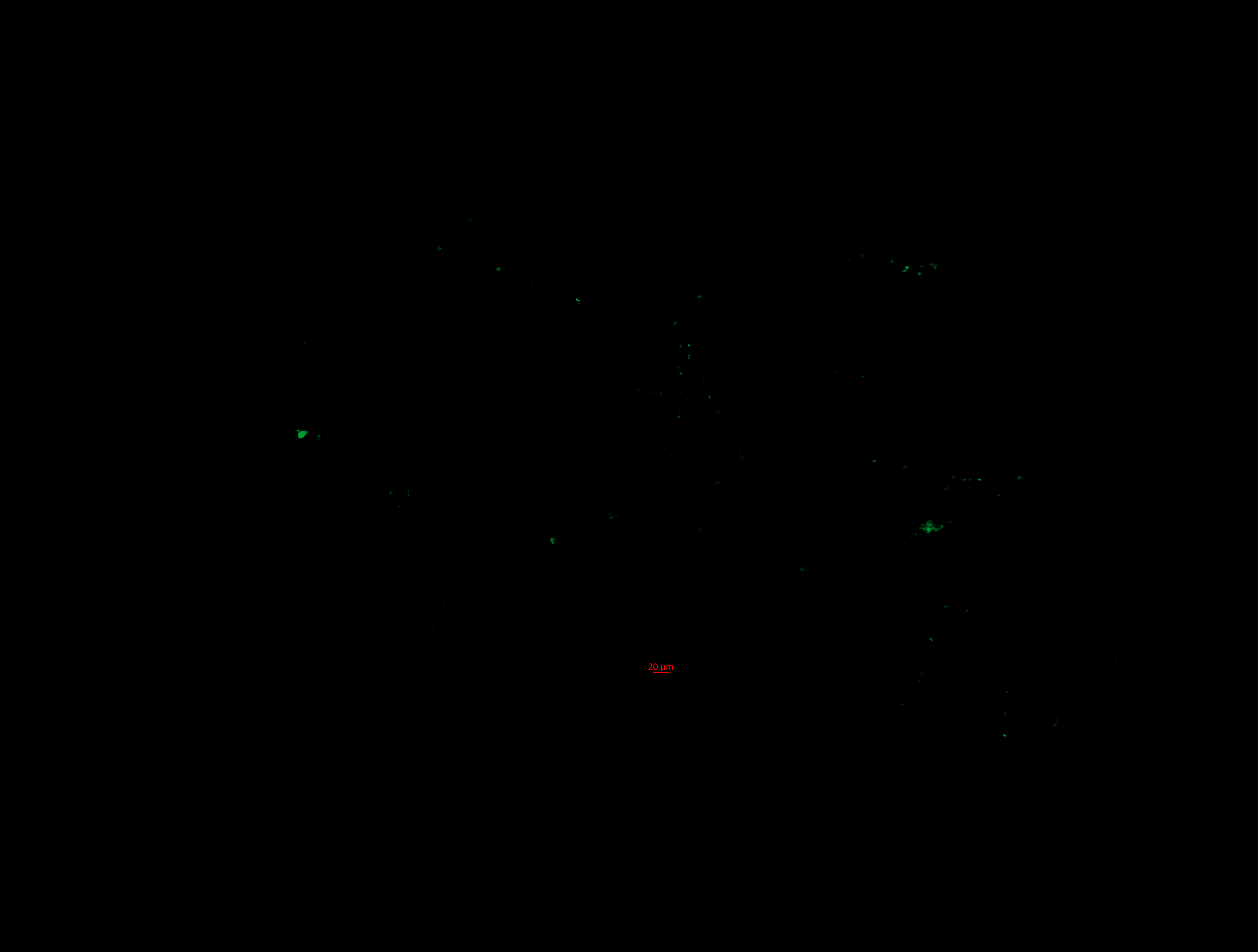

Supplement: Supplementary file 6 [file Data_Sheet_2.ZIP › Image2/Figure 4B/E9.5/sp27-9-AGM-20x_c3.tif]

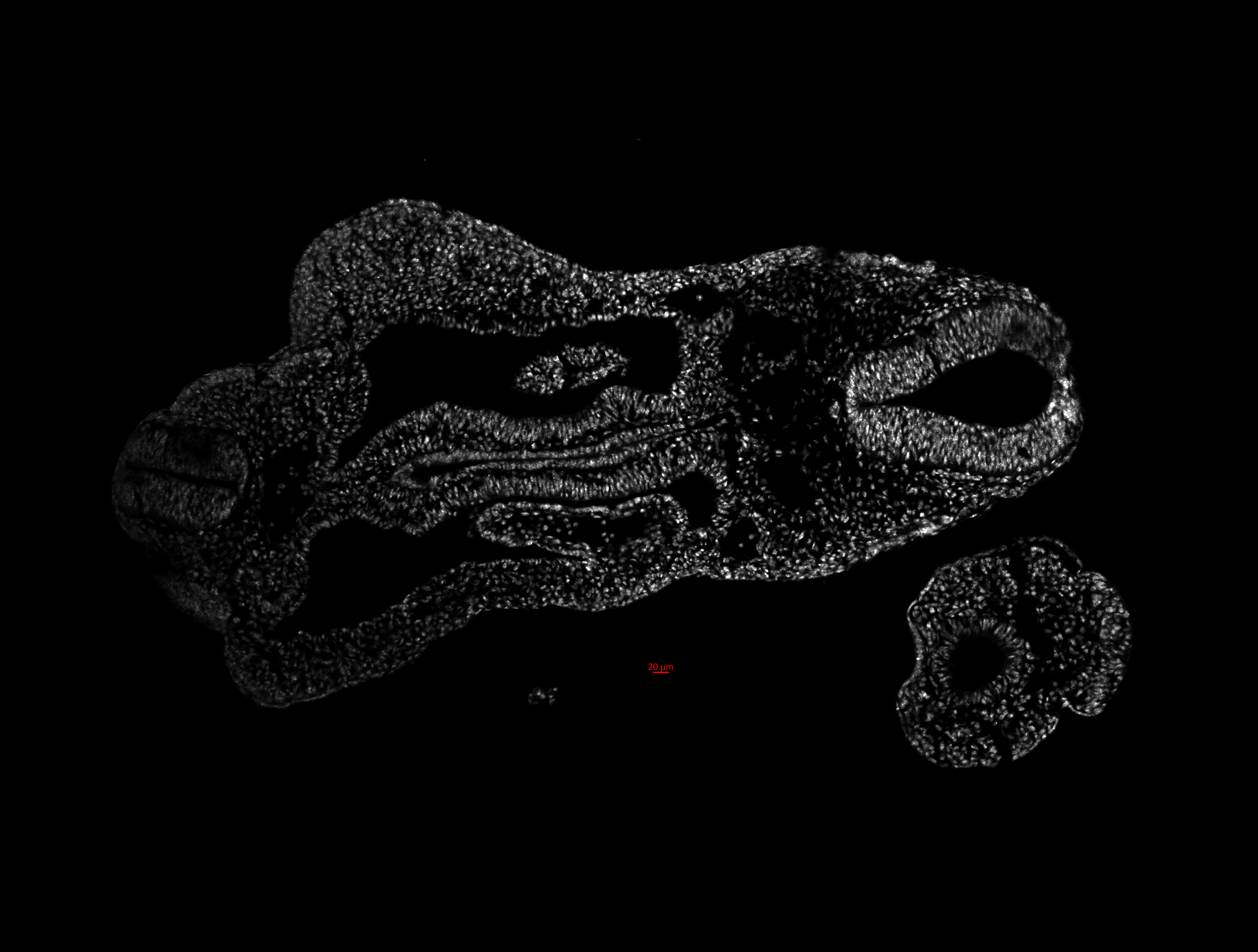

Supplement: Supplementary file 6 [file Data_Sheet_2.ZIP › Image2/Figure 4B/E9.5/sp27-9-AGM-20x_c4.tif]
